# Who were the miners of Allumiere? A multidisciplinary approach to reconstruct the osteobiography of an Italian worker community

**DOI:** 10.1371/journal.pone.0205362

**Published:** 2018-10-11

**Authors:** Marica Baldoni, Gabriele Scorrano, Angelo Gismondi, Alessia D’Agostino, Michelle Alexander, Luca Gaspari, Fabrizio Vallelonga, Antonella Canini, Olga Rickards, Cristina Martínez-Labarga

**Affiliations:** 1 Laboratorio di Antropologia Forense e Biologia dello Scheletro, Dipartimento di Biologia Università degli Studi di Roma “Tor Vergata”, Roma, Italia; 2 Laboratorio di Medicina Legale, Dipartimento di Biomedicina e Prevenzione, Università degli Studi di Roma “Tor Vergata”, Roma, Italia; 3 Centro di Antropologia Molecolare per lo Studio del DNA antico, Dipartimento di Biologia Università degli Studi di Roma “Tor Vergata”, Roma, Italia; 4 Laboratorio di Botanica, Dipartimento di Biologia Università degli Studi di Roma “Tor Vergata”, Roma, Italia; 5 Department of Archaeology, BioArCh, University of York, York, United Kingdom; 6 Sapienza Università di Roma, Dipartimento di Scienza dell'Antichità, Cattedra di Archeologia Cristiana e Medievale, Roma, Italia; University of Florence, ITALY

## Abstract

This research presents an in-depth study of the skeletal remains collected from the archaeological site of Allumiere (15^th^-16^th^ centuries CE; Rome, Italy). A multidisciplinary approach was used, combining skeletal biology, molecular anthropology and archaeobotany with the aim of reconstructing the osteobiography of the alum miners buried at the site. Since 1460, the area of the Tolfa Mountains was significant for the exploitation of alum which was used for a wide range of purposes in the Middle Ages, ranging from woven production to medical practice. A total of 70 individuals (63 adults and 7 juveniles) were studied. The sex ratio of the community indicated a higher prevalence of males with respect to females. Morphological examination indicated occupational musculoskeletal stress markers, which might reflect the specific phase of alum production that each individual was occupied in. Dietary reconstruction was primarily performed through carbon and nitrogen stable isotope analysis with integration of the results obtained by microscopic, genetic and GC-MS investigations on dental calculus. The diet was omnivorous, indicating a reliance on C_3_-terrestrial protein and evidence for limited C_4_ consumption by some individuals. Herbivores, such as sheep and cattle, appear to have contributed to the diet more than pigs and chickens. Consumption of Fagaceae and Poaceae species was predominant; moreover, indicators of Brassicaceae and milk and its derivatives were abundantly recurrent in the population, followed by plant oils and theophylline. Furthermore, the detection of pharmacological alkaloids indicated the knowledge and application of medicinal plants by the community. The novel use of multiple techniques based on cutting-edge technologies has provided a unique window on the lifestyles of individuals from one of the first Italian settlements of alum workers.

## Introduction

The exploitation of alum in Italy started in 1460, when Giovanni di Castro, a commissioner of the Pontifical State, identified the presence of alunite, a mineral from which alum could be extracted, in the territory of the Tolfa Mountains (Rome, Italy) [1–3]. Alum is a salt made up of ammonium sulfate and potassium associated with twenty-four molecules of crystallization water, whose applications ranged from textile production to medicine. Because of its water solubility, alum is not directly available in nature, but it is obtained through transformation of the less soluble aluminum minerals, such as alunite. The discovery of alunite on the Tolfa Mountains was of vital importance for the papal coffers and influenced the rise of the Western European textile industry. This source became particularly important after the conquest of Constantinople by the Turks in 1453 meaning that alunite deposits located in the Eastern Mediterranean area became difficult to access [2]. The extraction of the alum was entrusted to companies of contractors while the Apostolic Chamber handled its marketing. From the beginning, the Medici family secured the control of commercialization of the product in collaboration with Genoese merchants. In 1499, the banker Agostino Chigi was responsible for the organization of the mining enterprise and settlement of the area and the birth of the village that would later become Allumiere, is likely down to his actions [2].

Extraction and processing of the alum required the involvement of specialized manpower. The production cycle of alum, described in detail by historical sources [1, 3], began with the excavation of the mineral from the rocks through the use of picks. Then, alunite stones were heated at high temperature (i.e. 600–700°C) in special furnaces for 12–14 hours. The "roasted" mineral was finally treated with water, in order to obtain a doughy solution, which was then heated again and concentrated until alum crystals began to separate.

**Fig 1:**
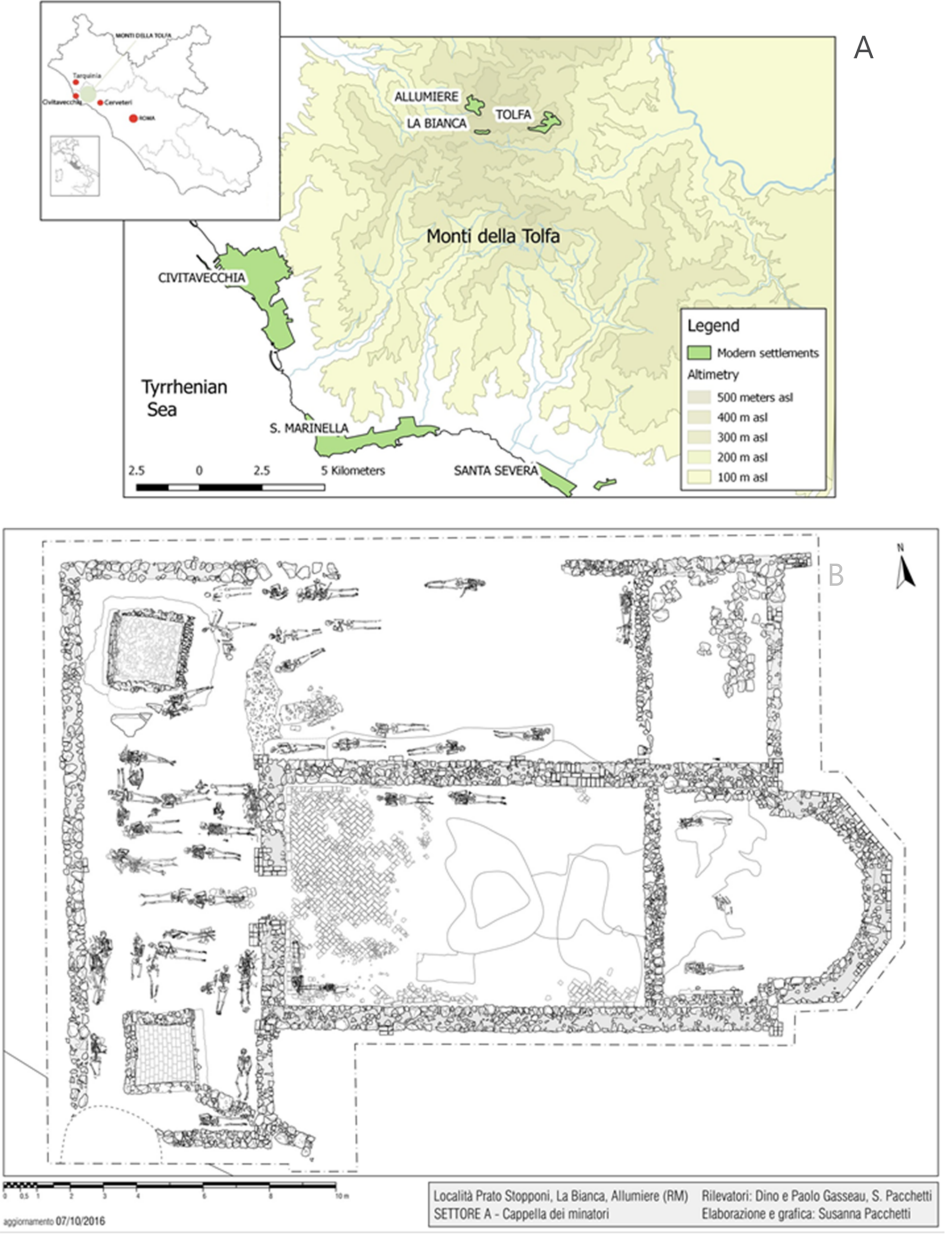
A) Map of the geographical area of *La Bianca* B) archaeological plan of the church named *Cappella dei Minatori* with the position of the skeletal remains.

In the area of *La Bianca*, several excavation campaigns have unearthed a Medieval cemetery and a church named *Cappella dei Minatori* (Fig 1). It has a single nave, is East-West oriented and is 19 m long and 8 m wide. The structure and dating of the church suggest that the archaeological site was related to one of the first human settlements in the area associated with alum production. A total of 70 burials were found, most of them located outside the church (Fig 1).

Archaeologically, the stratigraphic sequence of the site is challenging to define, as is common in Medieval cemeteries. In the majority, of the taphonomic observations, the burials were made up of simple earthen graves with individuals wrapped in shrouds, with the exception of a wooden coffin identified through the presence of *in situ* nails (SU 296). A chronological sequence of burials was indicated by earlier burials were being laid in a N-S orientation and subsequent later individuals buried on an E-W axis.

Items of jewelry and coins were recovered with a few individuals. In SU 110, three silver coins were found close to the left hand; SU 135 was buried with a religious medal, portraying the Immaculate Conception and SU 245 had eleven gold and two silver coins close to the left hand. All these artifacts confirmed the dating of the cemetery; in particular, the silver coins of SU 110 were dated to 1476–1483 [4]. Further archaeological analyses revealed various origins for these coins. Some coins possessed a “fleur-de-lis”, symbol of the city of Florence (Italy). Others were also identified as three ducats of Ferdinando I from Naples, one coin from the Pontifical State and three from Venice. Others were of Iberian origin, specifically, a *doble excelente de la Granada* and an *excelente* of the kings Fernando de Aragon and Isabel de Castilla and a Portuguese *cruzado* of King Giovanni II d'Aviz. There was also a duchy from Rhodes. The dating of the coins ranged between 1464–1523. The variety of coins and their origins are an important proof of the wealth of commercial traffic linked to alum.

The present research aims to reconstruct the osteobiography of these alum miners of the Tolfa Mountains through a multi-proxy approach, combining skeletal biology, molecular anthropology and archaeobotany. Osteological analysis allows the reconstruction of the biological features of the Medieval workers, while molecular and archeobotanical analyses are used to shed light on the dietary patterns and medicinal habits of the community of Allumiere.

There is a general lack of information about the dietary habits of the lower social classes in historical texts from the Medieval period. Therefore, to better understand the dietary pattern of the studied community, we applied carbon (δ^13^C) and nitrogen (δ^15^N) stable isotope analysis to human and animal bone collagen. Stable isotope analysis of bone collagen represents an established technique to identify the main protein sources in the diet of archaeological populations. This allows us to investigate marine and terrestrial and C_3_ and C_4_ sources of protein in addition to the trophic position an individual is feeding at [5–7]. Analysis of dental calculus was also carried out to further investigate which vegetal and animal species were present in miners’ diet. Indeed, the analysis of dental calculus is a very informative archaeobiological approach to reconstruct past food habits [8–10]. Dental calculus is a dense mineral matrix made up of inorganic salts and organic molecules derived from ingested foods, crystallized remains of oral microbiota and accidentally inhaled microremains [11–12]. Light microscopy, genetic and gas-chromatographic mass-spectrometry approaches were carried out on dental calculus to provide direct evidence of the main categories of foods and plant drugs introduced, at least once, in the lifetime of our ancient exemplars [13–23]. The present work provides new information on an important period of the Italian Medieval history, shedding light on the lives of those who lived in one of the first Italian settlement of alum miners.

## Materials and methods

The present research deals with the skeletal remains recovered in the area of La Bianca in Allumiere (Rome, Italy) (Fig 1A) in a graveyard close to the church named as *Cappella dei Minatori* (Fig 1B). The archaeological excavation started in 2010 directed by Dr. Fabrizio Vallelonga authorized by the “Comune di Allumiere” (Municipality of Allumiere). The research was carried out at the Department of Biology of the University of Rome “Tor Vergata” and directed by Dr. Cristina Martínez-Labarga who received the authorization for the analysis of the skeletal remains from La Bianca (Allumiere, Rome, Italy) in 2015. The complete list of the specimens is provided in S1 Table.

### Osteological examination

Seventy individuals were analysed, both adults and non-adults collected from the cemetery area of *La Bianca* (Allumiere, Rome, Italy). The preservation index for the individuals was calculated following the method proposed by Walker et al. [24]. The age estimation for adult individuals (from ca. 18 year old) followed methods based on morphological changes in the pubic symphysis [25–27], in the auricular surface of the ilium [28] and in the sternal end of the fourth rib [29–30]. Secondarily, dental wear [31–32] and obliteration of the cranial sutures [33] were also observed. Age estimation for infant and juvenile skeletal remains (until ca. 18 years old) was carried out through the diaphyseal length of bones [34–36], and tooth eruption [37]. Moreover, secondary ossification centers were taken into account, following the methods proposed by different authors and summarized in Minozzi and Canci [38].

Sex diagnosis was performed only on adult samples, as proposed by Acsàdi and Nemeskèri [39] and revised by Ferembach et al. [40] and Phenice’s [41]. Metric analysis complemented the morphological data, as the state of preservation of the sample allowed the measurement of sexually dimorphic bones, utilizing univariate and multivariate techniques [42–46].

Osteometrics was applied following the methods and standards proposed by Martin and Saller [47] and Borrini [48–49]. Cranial and post-cranial indices were calculated following the guidelines proposed by several authors [38, 43, 50–51]. Living stature was estimated as the average value obtained using multiple methods [52–56]. When skeletal remains were fragmented, Steele’s formulae [57] were applied to estimate long bone length. Moreover, working activities were determined analyzing enthesal changes (EC) as proposed by Mariotti et al. [58–59] and by Borgognini Tarli and Reale [60]. The paleopathological survey was performed through morphological observation of the skeletal remains. In one pathological case, X-ray monitoring was carried out at the “Dipartimento di Diagnostica per Immagini, Imaging molecolare, Radiologia, Interventistica e Radioterapia, Azienda Ospedaliera Universitaria Policlinico Tor Vergata”, using GMM Opera Swing, in order to achieve a differential diagnosis.

Particular attention was paid to evidence of osteoarthritis, which was scored according to the standards proposed by Buikstra and Ubelaker [61]. These authors evaluated intensity and distribution of lipping, porosity and eburnation of the osteoarthritis. A scale system from 0 -absence of injury on bone tissue- to 3 -marked modification of the joint surface- was used. In order to better evaluate the position in which the lesion occurred, Prieto’s joint division scheme was adopted [62]. This method distinguishes proximal and distal epiphyses, dividing them in four areas: two superiors (anterior and posterior) and two inferiors (anterior and posterior).

Statistical analyses were carried out using the statistical software R (v. 3.4.1) for the Student’s t-test and chi square test with Yates correction.

### Isotope analyses

Collagen extraction was carried out on 68 human and 12 faunal skeletal remains from the archaeological cistern (1 *Hystrix cristata*, 1 *Cervus elaphus*, 2 *Equus asinus*, 2 *Felix catus*, 1 *Canis lupus* 2 *Sus domesticus*, 1 *Equus caballus* and 2 unidentified carnivores) in order to conduct carbon and nitrogen stable isotope analysis. Human samples comprised of rib bones, whereas animal samples were taken from the various skeletal elements that were available.

Collagen extraction followed a modified Longin method [63]. Initially, to remove potential contaminants a sterile surgical blade was used on the outer surface of the bone samples and c. 500 mg of bone was subsequently pulverized using a mortar and pestle. To demineralize the bone, 8 mL of HCl 0.6 M at 4°C was added to the powder and left at 4°C on a horizontal mixer for two days, changing the acid after 24 hours.

Once the mineral component of the bone removed, samples were rinsed three times with ddH_2_O, until the pH level became neutral. The resultant pellet was gelatinized at 75°C, for 24–48 hours, with HCl pH 3.0 (0.001 M). The solution was then frozen at -80°C for four hours and then freeze-dried for two days. A simultaneous extraction on modern bovine bone was performed and used as reference control. Approximately 0.8–1.2 mg of collagen was weighed and analysed in duplicate by EA-IRMS on a Sercon GSL analyser coupled to a Sercon 20–22 Mass Spectrometer at the University of York. The analytical error, calculated from repeated measurements of each sample, an internal laboratory control (fish gelatine), and international standards, was <0.2‰ (1σ) for both δ^13^C and δ^15^N. International Atomic Energy Agency (IAEA) standards were N-2, and 600 for nitrogen and International Atomic Energy Agency IAEA-600 and Iso-Analytical R006 for carbon. Isotope data are reported as delta (δ) values relative to V-PDB (Vienna Pee Dee Belemnite) for carbon and AIR (Atmospheric air) for nitrogen. Carbon content (%C), nitrogen content (%N), protein yield, and C/N ratios were checked to monitor the diagenesis of bone [64] and determine bone protein quality for paleodietary reconstruction, according to DeNiro [65] and van Klinken [66].

### Molecular and archaeobotanical analyses on dental calculus

#### Sample collection and decontamination

Dental calculus study was performed on 35 individuals, who were the only ones presenting maxillary and mandibular bones and teeth. Each analysis was carried out on different aliquots of calculus. Sodium hypochlorite 5% and UV exposure were employed to treat tools and working surfaces to restrict contamination during the analysis. In addition, different laboratories were used to carry out the various phases of this work [67]. Deposits of dental calculus were generally slight, according to Brothwell [31], on all dentition. Initially, to eliminate any contamination before sampling, a scraping action was applied by a sterile surgical blade on the outer surface of the dental calculus. All collecting procedures were carried out under a sterile vertical laminar flow hood (Heraeus HERAsafe HS12 Type). In order to remove any environmental contaminants from the calculus surface, samples were UV-treated for 10 minutes on each side and soaked in 5% sodium hypochlorite for 15 minutes. Lastly, the calculus was washed in sterilized bidistilled water and rinsed in 100% ethanol to eliminate the aqueous components before being left to dry [22, 68]. To validate sterilization protocols, five dental calculus samples previously subjected to decontamination procedures, were randomly selected and washed with 200 μL of water. These last washing solutions were subjected to light microscopy, genetic and GC-MS analysis. No microremains, nucleic acid or chromatographic signals were detected, confirming the efficacy of the cleaning methods.

#### DNA extraction, amplification and sequencing

All criteria and precautions for the study and analysis of ancient DNA (aDNA) were applied [69–71]. For aDNA extraction a modified protocol suggested by Warinner and collaborators [10] was used. For each sample (50 mg of pulverized dental calculus), 600 μL of extraction buffer (100 mM Tris-HCl pH 8, 100 mM NaCl, 10 mM EDTA and 2% SDS) and 50 μL of proteinase K (20 mg/mL) were added. The samples were incubated in a shaking water bath at 56°C, for 6 hours, adding 20 μL of fresh proteinase K (20 mg/mL) every 2 hours. The sample was then incubated overnight at 37°C. Following this, the sample was centrifuged for 5 minutes at 13.000 rpm and the supernatant was transferred to a new 2 mL Eppendorf tube. The supernatant was mixed with 500 μL of phenol/chloroform/isoamyl alcohol (25:24:1) and, after a centrifugation of 5 minutes at the 13.000 rpm, transferred into a new 15 mL falcon tube. DNA was purified by QIAquick PCR purification kit following the manufacturer’s procedure, and eluted into 50 μL of the elution buffer. Ancient DNA extracts were stored at 4°C.

For each sample, different polymorphic regions of mitochondrial genome of several animal species were amplified (35 PCR cycles), using pairs of primers that only amplified for the target species (S2 Table). For ovine and pig, the COX 1 gene was amplified, while 12S rRNA and 16S rRNA were analysed for bovine and chicken, respectively. These primers were proposed by Natonek-Wiśniewska and colleagues [72]. For fish investigations, the DNA Mini-Barcoding System reported in Shokralla and collaborators [73] was applied. Amplifications were also performed for negative and positive controls. Amplifications of modern and ancient DNA were performed separately in two different laboratories. In particular aDNA analysis was carried out in the aDNA Laboratory in the Departmental Center of Molecular Anthropology for Ancient DNA Studies, University of Rome, Tor Vergata, in Villa Mondragone, Monte Porzio Catone, Rome (http://www.bio.uniroma2.it/biologia/laboratori/lab-antropologia/DNAantico/DNA_antico/Facilities.htm) which has all the facilities to minimize potential contamination with extant DNA [71]. In order to detect the correct activity of the different reagents, the positive controls that contained modern faunal DNA were prepared in the laboratory for modern DNA processing in the department of Biology, of the University of Rome “Tor Vergata”.

To verify the amplifications, PCR products were separated by electrophoresis on 1.5% agarose/Tris-acetate EDTA gel and the amplicons were then purified by enzymatic digestion with HT ExoSAP-ITVR (Affymetrix) following the manufacturer’s procedure and sequenced using the same PCR primers by an ABI PRISM 3130 Genetic Analyzer (Applied Biosystems, Foster City, CA, USA), as reported in Gismondi et al. [74]. The BLAST website tool (https://blast.ncbi.nlm.nih.gov/Blast.cgi?PROGRAM=blastn&PAGE_TYPE=BlastSearch&LINK_LOC=blasthome) was used to confirm the identity of the sequences found in the dental calculus. The presence of fish DNA was detected by polyacrylamide gel, using a molecular weight marker (GelPilot 50bp Ladder Qiagen).

#### Light microscopy analysis (LM)

A protocol based on Hardy et al. [75] method was used to extract starch granules and other microremains from dental calculus. Twenty mg of each sample were resuspended in 500 μL of 1 M HCl and sonicated for 10 minutes (Falc Instruments MOD: LBS1 34) before being left under agitation for 24 h. After centrifugation at maximum speed for 10 minutes, the pellet was subjected to 3 consecutive washings with bidistilled water. The last pellet was resuspended in 100 μL of bidistilled water, sonicated for 5 minutes and observed at OM (Nikon Eclipse E100), under white and polarized light. The whole volume of the sample was examined and microfossils were photographed at 100X magnification using software for capturing images (ProgRes CapturePro 2.9.0.1) and measured (i.e. for starch granule, maximum length through the *hilum* and maximum width anywhere along the perpendicular *axis*) by the SuperAmpelo 2.0 program. Taxonomic identification of pollens, phytoliths and starch granules was carried out by direct comparison with a modern experimental collection (hosted in Laboratory of Botany and Botanical Gardens of the Department of Biology at University of Rome “Tor Vergata”) or data from literature, including a pollen Atlas [76].

#### Gas-chromatography mass-spectrometry (GC-MS) analysis

This analysis was carried out in qualitative and non-quantitative terms, according to Gismondi et al. [77]. Ten mg of calculus for each individual were dissolved in 1 mL of 6% HCl. Once left in agitation for three days, 1 mL of hexane was added and shaken for two hours. After centrifugation for 10 minutes at 10.000 rpm, the supernatant hexane fraction was recovered and dried by a speed-vac system (Eppendorf AG 22331 Hamburg, Concentration Plus). The dried pellet was then derivatized by resuspension with 50 μL of hexane and 50 μL of the Methyl-8-Reagent (Thermo Scientific), in thermostated bath, at 60°C for 20 minutes. At least 3 analyses for each sample, by injecting 2 μL of extract in GC-MS (QP2010, Shimadzu, Japan; column DB-5 Phenomenex; helium as gas carrier; splitless modality), were carried out. The run was conducted by a temperature gradient: initial oven temperature was set at 60°C for 5 min; then, increasing temperature at a fixed rate of 6°C per minute, the column was heated up at 150°C for 5 minutes, 250°C for 5 minutes and 330°C for 25 minutes. Parameters and conditions relating to mass spectrometry were: ion source temperature 230°C; interface temperature 320°C; solvent cut time 6 minutes; ionization mode: EI; ionization voltage: 0.70 eV. The identification of each molecule (similarity values were considered acceptable only if higher than 85%) was performed comparing their mass spectrum with those registered in the NIST Library 14 loaded on detection software. No significant differences among replicates were detected.

## Results

### Skeletal biology

The present series of Medieval samples consisted of 70 individuals, 63 adults and 7 non-adults, sampled from primary burials in the cemetery area of *La Bianca*. The preservation index was calculated for each skeleton (ranging from a minimum of 0 to a maximum of 93%), showing an average preservation value of 59.4%. Individuals were sorted into 9 age classes. The composition by sex and age at death is summarized in Table 1. It is worth pointing out the under-representation of sub-adults with respect to adults and the high prevalence of males with respect to females among the adults. The imbalance between sexes is indicated by the elevated sex ratio (M:F = 8.0) which is significantly different from the range of expected proportions (χ^2^, p<0.05).

**Table 1:** Sex and age at death determination in the analyzed series from Allumiere (Rome. Italy).

AGE GROUP	Male	Female	Indefinite*	Not-Recordable**	Indeterminate***	Total (% of total population)
< 1 year	0	0	0	0	2	2 (2.9%)
Infant I 1–6 years	0	0	0	0	1	1 (1.4%)
Infant II 7–12 years	0	0	0	0	3	3 (4.3%)
Adolescents 13–18 years	0	0	0	0	1	1 (1.4%)
Young Adult 19–30 years	17	2	0	0	0	19 (27.1%)
Adult 31–40 years	19	2	2	0	0	23 (32.8%)
Mature 41–50 years	8	1	0	0	0	9 (12.9%)
Senile > 50 years	3	0	0	0	0	3 (4.3%)
Indeterminable Adult (IA) 19-x years	1	1	0	7	0	9 (12.9%)
Total **(% of total population)**	48 (68.5%)	6 (8.6%)	2 (8.6%)	7 (10.0%)	7 (10.0%)	70 (100%)

*Indefinite indicates a mixture of male and female traits;

**Not-recordable indicates the impossibility to perform sex and age at death diagnosis;

***Indeterminate indicates non-adults for whom no assumption of sex was performed.

For adult individuals, the highest mortality falls between the young adult and adult classes. Data on the juveniles’ mortality rate data should be considered cautiously due to the small sample size.

Living stature was estimated on 43 individuals (37 males and 6 females). The mean stature for males was 169.22 ± 6.26 cm, while females exhibited an average stature of 156.70 ± 5.96 cm. A Student’s t-test indicated a statistically significant difference between the sexes (*p*-value = 3.17x10^-7^). Post-cranial indices revealed high skeletal robusticity and skeletal asymmetry in males. In particular, the right side seemed to have been subjected to a higher biomechanical stress than the left. The results obtained for all the indices are shown in S3 Table. Pathological assessment was carried out on 57 adult individuals (S4 Table). Six individuals were excluded due to poor preservation.

**Fig 2:**
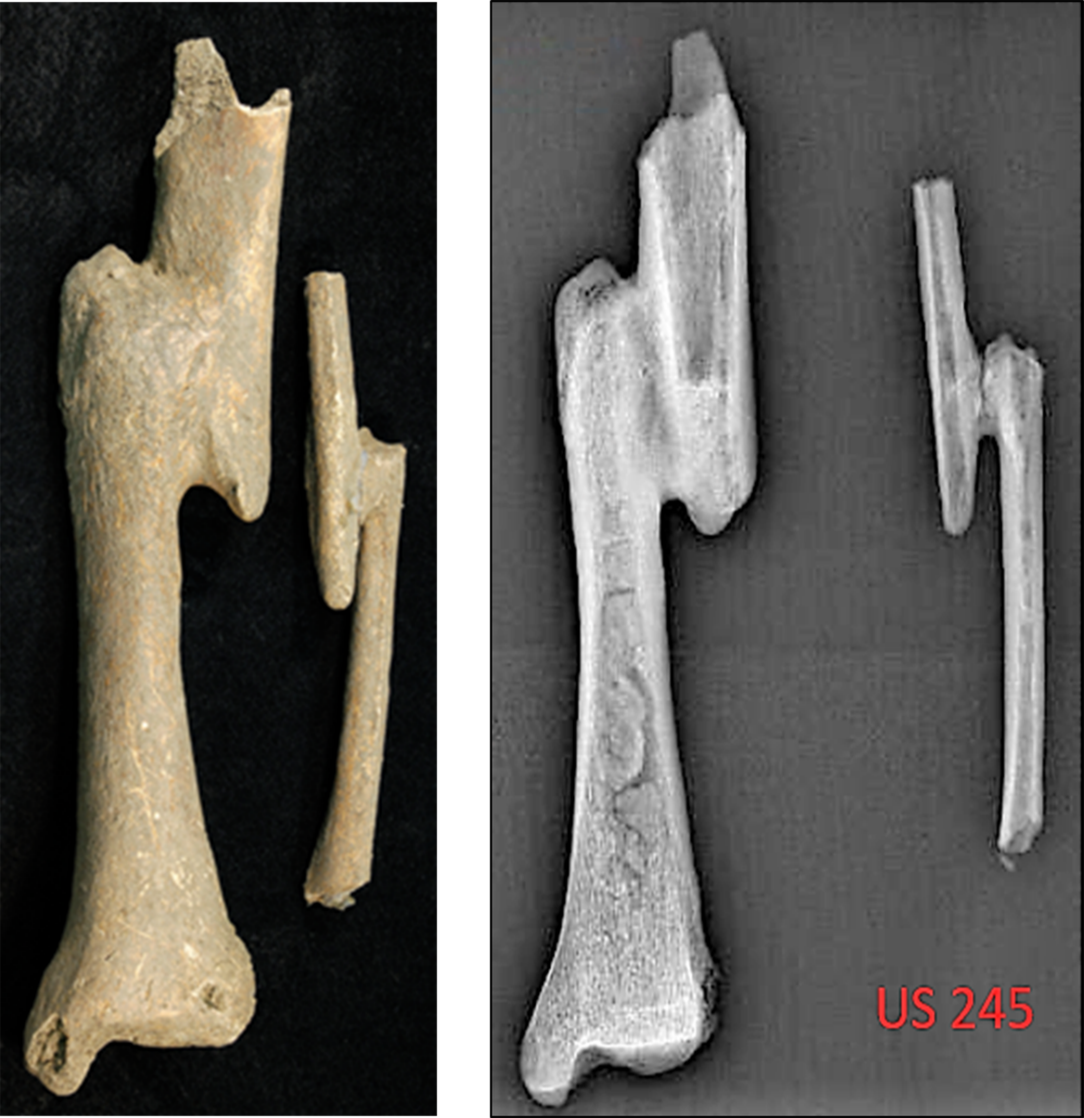
*Ante-mortem* compound fracture affecting SU 245. A) compound fracture on both tibia and fibula; B) X-rays analysis (X-ray exposure: 78 kV).

The highest frequencies were recorded for degenerative diseases (86%), Schmörl’s nodules (75%), periostitis (32%), and fractures (27%). Degenerative diseases were considered, jointly with enthesal changes, able to associate each individual to the most plausible phase in alum production. Fractures were relatively common within the community. For example, SU 245 demonstrated an *ante-mortem* compound fracture which affected both tibia and fibula. This trauma (Fig 2) caused the loss of ca. 5 cm in limb length.

The analysis of musculoskeletal stress markers was conducted on male individuals as historical evidence indicates [1,3] that they would have been involved in alum extraction and production. No detection attempt was made on juveniles because the high plasticity of their skeletal remains could provide unreliable results. Only 20 male specimens were considered suitable for all further analyses on enthesal changes. The remainder were excluded either due to their state of preservation, which prevented macroscopic observation of muscular insertion sites, or because, as was the case for SU 245, a trauma or skeletal disease was present that could alter interpretation. Indeed, the musculoskeletal stress markers of SU 245 seemed to be influenced by skeletal lesions. The enthesal changes observed in the upper limbs of this individual (i.e. a greater muscle development on the right humerus than on the left) led us to hypothesize that they would have used a crutch to partially solve the walking difficulty caused by the loss of the original length of the limb as consequence of the *ante-mortem* fracture.

On the basis of the development of occupational stress markers, the individuals seemed to cluster in four groups, corresponding to the four different phases of alum production (excavation, calcination, moistening and lixiviation) [78]. These data were also corroborated by the results of the post-cranial indices, which revealed different patterns of skeletal strength as well as a functional asymmetry. Musculoskeletal stress data were finally integrated with the analysis of degenerative pattern and joint diseases. Osteoarthritis could be observed on 45 samples (S5 Table).

Osteoarthritis confirmed all previous suppositions about musculoskeletal stresses and alum working activity of the individuals. The excavation phase caused a marked enthesal development of shoulders, elbows, hips and feet; in the calcination the most involved functional groups are shoulder, elbow, hip and knee; the individuals involved in moistening exhibit a high degree of biomechanical stress that affected shoulders, hips, knee and feet, while those employed in lixiviation show a great muscle development in the shoulders, forearms, hip and foot.

All the working phases are characterized by well-developed musculoskeletal markers on the shoulders and the hip however, the combination of other functional groups significantly diverges amongst the other clusters. For example, the skeletons that show high robusticity of *m*. *pectoralis major* (horizontal flexion of the upper limb), *m*. *deltoideus* (abduction, extension and flexion of the limb) and *m*. *biceps brachii* (flexion of the forearm on the arm) could be related to the excavation phase according to the upper limb’s movements involved in the use of the picks. Even if, as mentioned, all phases of alum production cause a high biomechanical stress on muscles, the movement related to each one as well as the functional groups involved are different, and the excavation was observed to be the most strenuous phase involving the strain on both the shoulder and the elbow [1, 3].

Furthermore, a high degree of biomechanical stress on the knee could be observed in both moistening and calcination phases. However, moistening should involve a higher robusticity of the *quadriceps tendon*, whereas in the calcination *m*. *vastus medialis* should appear more distinctly stressed. The combined results of musculoskeletal stress markers and degenerative diseases are provided in Table 2.

**Table 2:** Combined results of musculoskeletal stress markers and osteoarthritic pattern (nd: not determinable).

INDIVIDUAL	SEX	AGE AT DEATH	ENTHESAL DEVELOPMENT	DEGENERATIVE PATTERN	HYPOTHESIZED ACTIVITY
**139**	M	41–50	shoulder/knee	shoulder/ankle/foot	moistening
**144**	M	31–40	shoulder/hip/knee	shoulder	moistening
**147 Aa**	M	19–30	shoulder/hip/foot	shoulder/elbow/hip	lixiviation
**159**	M	31–40	shoulder/elbow/hip	shoulder	lixiviation
**169**	M	19–30	shoulder/foot	shoulder/wrist	moistening
**176**	M	19–30	shoulder/forearm/hip /foot	shoulder/elbow/hip	lixiviation
**185**	M	51–60	hip/foot	shoulder/wrist/hip/knee	lixiviation
**189**	M	19–30	hip/knee/foot	shoulder/wrist/hip/knee	moistening
**198**	M	31–40	shoulder/elbow/hip/foot	elbow/wrist/hip/ankle	excavation
**221**	M	31–40	nd	shoulder/elbow/wrist/hip/ knee	calcination
**231**	M	41–50	nd	shoulder/elbow/wrist/hip	excavation
**277**	M	41–50	nd	shoulder/elbow/hip/knee	calcination
**280**	M	41–50	shoulder/elbow/hip/ knee	elbow/hip	calcination
**296**	M	51–60	high biomechanical stress	shoulder/elbow/wrist/hip	excavation
**303**	M	19–30	shoulder/elbow/hip/foot	shoulder/elbow/hip/ankle	excavation
**307**	M	31–40	shoulder/hip/knee/foot	shoulder/hip	moistening
**318**	M	19–30	shoulder/foot	shoulder/ankle/foot	moistening
**319**	M	19–30	shoulder/foot	shoulder/hip/foot	moistening
**320**	M	31–40	forearm/hip/foot	hip	lixiviation
**330**	M	41–50	shoulder/forearm/hip/ knee/foot	shoulder/wrist/hip	excavation
**362**	M	31–40	shoulder/elbow/hip/foot	shoulder	excavation

Unfortunately, a clear relationship between musculoskeletal markers, osteoarthritis and performed working activity could be identified only for twenty-one individuals because the others exhibited musculoskeletal stress markers or osteoarthritis patterns that could made them suitable for more than one working phase.

### Stable isotope analysis from bone collagen and molecular analysis from dental calculus

Isotopic data and quality indicators are presented in Table 3.

**Table 3:** Carbon and nitrogen stable isotope values and collagen quality indicators of animals and humans from *La Bianca*. The samples that fell outside the quality range parameters are indicated in red and were excluded from subsequent analysis.

**Human samples**								
**SU**	**sex**	**Age at death** **(years)**	δ^**13**^**C**	δ^**15**^**N**	**% C**	**% N**	**C:N Ratio**	**Collagen yield**
110	M	41–50	-17.6	8.1	38.3	13.4	3.3	2.4
135	M	31–40	-19.2	7.9	41.9	15.5	3.2	3.0
139	M	41–50	-20.0	8.1	29.6	10.0	3.5	5.9
144	M	31–40	-19.2	10.0	39.1	14.1	3.2	3.3
147	M	19–30	-18.7	9.6	40.9	15.0	3.2	2.0
158	M	31–40	-18.7	9.4	43.9	16.1	3.2	6.4
159	M	31–40	-20.1	9.0	30.1	10.1	3.5	4.3
169	M	19–30	-18.8	9.7	41.8	15.2	3.2	13.8
173	M	19–30	-19.6	6.7	43.2	15.6	3.2	11.0
176	M	19–30	-17.9	8.2	40.7	14.7	3.2	6.0
179	M	31–40	-21.2	7.8	16.3	4.8	3.9	0.8
182	ND	31–40	-17.1	7.2	40.7	15.1	3.2	2.7
185	M	51–60	-19.5	9.7	39.8	14.1	3.3	2.8
189	M	19–30	-20.1	6.2	36.8	13.3	3.2	3.4
192	M	19–30	-16.4	6.8	40.8	14.8	3.2	8.6
195	M	IA	-18.1	8.1	39.9	14.5	3.2	2.4
198	M	31–40	-28.1	-0.6	17.8	4.5	4.6	0.4
201	M	31–40	-18.4	7.7	37.7	13.5	3.3	1.0
204	M	31–40	-20.4	7.2	39.4	13.2	3.5	2.9
210	ND	IA	-20.4	9.1	24.0	8.2	3.4	1.9
213	ND	31–40	-19.4	10.8	39.4	12.7	3.6	2.0
216	F	41–50	-19.0	10.0	37.3	13.6	3.2	1.0
221	M	31–40	-18.8	9.1	40.0	14.1	3.3	7.3
226	ND	13–18	-17.6	8.2	41.8	15.0	3.3	2.2
231	M	41–50	-20.4	8.8	35.3	12.4	3.3	1.7
234	F	31–40	-20.0	8.8	39.3	14.2	3.2	2.1
239	M	19–30	-19.1	10.0	41.3	14.7	3.3	6.8
245	M	51–60	-18.5	12.1	39.1	13.6	3.4	3.0
249	M	19–30	-20.6	8.1	36.8	13.1	3.3	1.8
256	M	19–30	-19.2	7.7	41.6	14.7	3.3	5.8
269	M	19–30	-20.2	6.7	40.3	13.6	3.5	6.0
270	F	19–30	-19.1	9.3	41.0	15.0	3.2	3.8
272	M	31–40	-20.2	7.8	42.6	15.4	3.2	5.7
274	M	19–30	-19.8	7.2	40.4	14.3	3.3	1.4
277	M	41–50	-19.6	6.9	38.5	14.1	3.2	2.5
280	M	41–50	-20.6	8.8	38.8	13.8	3.3	1.2
284	ND	7–12	-19.4	9.3	40.8	15.0	3.2	4.5
290	M	41–50	-19.2	10.7	42.7	15.5	3.2	10.3
293	F	19–30	-20.3	10.4	38.0	13.3	3.3	0.8
296	M	51–60	-18.7	6.5	38.7	13.1	3.5	0.8
303	M	19–30	-19.8	10.9	32.2	11.0	3.4	0.8
304	ND	7–12	-19.3	9.4	41.3	15.0	3.2	2.86
307	M	31–40	-29.2	-1.8	9.0	2.4	4.3	0.6
308	M	19–30	-18.6	9.0	42.6	15.4	3.2	6.3
311	M	19–30	-20.1	7.9	39.0	14.2	3.2	5.0
317	ND	1–6	-18.8	11.9	41.8	14.9	3.3	3.2
318	M	19–30	-19.3	8.7	43.2	15.7	3.2	3.8
319	M	19–30	-17.2	8.2	43.5	15.7	3.2	4.0
320	M	31–40	-24.7	5.3	4.0	0.9	5.0	0.4
325	M	31–40	-24.9	4.2	3.8	0.7	6.0	0.5
330	M	41–50	-19.0	8.5	36.3	10.9	3.9	0.5
334	ND	7–12	-19.4	7.5	43.0	15.2	3.3	4.5
339	F	31–40	-19.6	7.2	40.1	14.6	3.2	2.9
343	M	31–40	-19.2	9.1	42.0	15.2	3.2	12.6
346	M	41–50	-19.3	9.9	43.3	15.4	3.3	4.8
356	M	31–40	-18.3	9.4	40.5	14.6	3.3	4.3
359	M	31–40	-17.9	9.2	38.3	13.7	3.3	3.0
362	M	31–40	-17.2	8.5	36.3	12.8	3.3	2.1
381	M	31–40	-19.9	7.5	35.5	12.2	3.4	1.0
382	ND	IA	-20.4	6.4	38.4	13.7	3.3	2.2
383	ND	IA	-19.7	10.1	29.4	10.2	3.4	1.1
385.3	ND	IA	-23.3	5.6	28.3	8.2	4.0	0.5
385.5	ND	IA	-21.0	7.4	19.6	5.5	4.2	1.6
385.6	ND	IA	-19.7	9.7	39.2	14.2	3.2	2.4
388	ND	IA	-20.5	5.9	39.2	14.1	3.2	1.6
416	ND	IA	-20.8	8.8	38.9	12.0	3.8	1.1
**Animal samples**								
**SU**	**species**		δ^**13**^**C**	δ^**15**^**N**	**% C**	**% N**	**C:N Ratio**	**Protein yield**
101 C A	*Hystrix cristata*		-20.6	3.6	38.6	13.3	3.4	2.7
168 C A	*Cervus elaphus*		-21.1	3.5	40.4	14.1	3.3	4.8
101 A	*Equus caballus*		-25.4	2.2	10.4	2.7	4.6	0.7
385 A	*Equus asinus*		-21.4	4.1	38.3	13.2	3.4	1.6
399.2 A	*Equus asinus*		-21.3	5.9	36.5	12.7	3.4	0.9
399 A	*Felix catus*		-20.1	7.5	39.6	14.1	3.3	2.8
101.3 A	*Felix catus*		-19.5	7.8	39.2	14.2	3.2	10.8
374 A	*Sus domesticus*		-21.8	4.7	41.6	13.9	3.5	3.5
355 A	*Sus domesticus*		-20.5	3.9	39.7	13.5	3.4	2.9
385.2 A	*Canis familiaris*		-20.1	7.5	39.9	14.1	3.3	2.3
101.4 A	*unspecified carnivorous*		-20.8	7.5	39.8	13.0	3.6	1.5
101.2 A	*unspecified carnivorous*		-19.9	8.5	40.6	14.7	3.2	8.5

**Fig 3:**
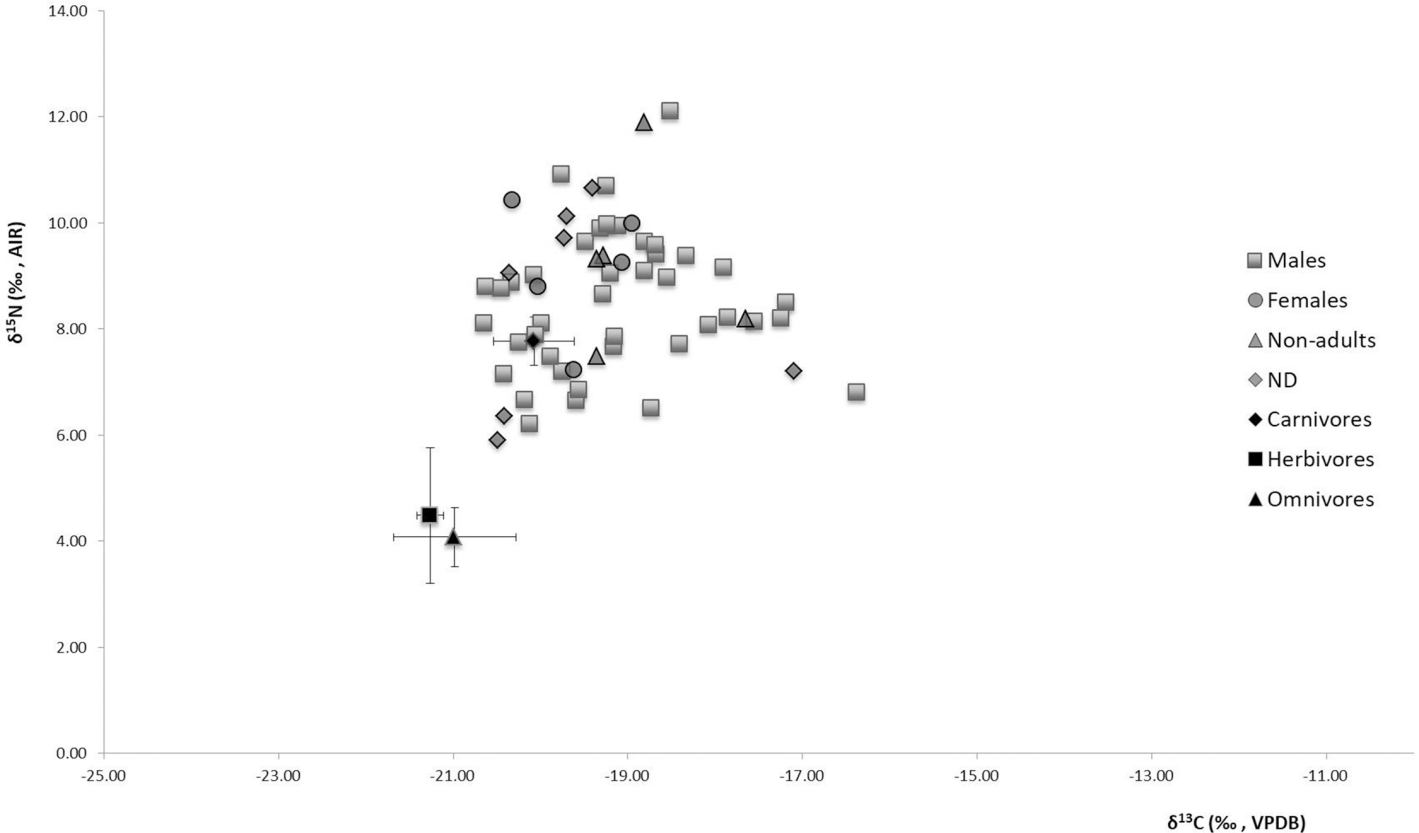
Plot of carbon *vs* nitrogen isotopic values for faunal and human remains from Allumiere. Human data are plotted individually and distinguished by age (adults and non-adults) and sex. Animal data are plotted as means and error bars represent ±1σ.

Ten humans (SU 213, 330, 198, 325, 320, 307, 179, 385.3, 385.5, 416) and one animal (SU 101 A *Equus caballus*), were excluded from the analysis due to their C/N ratio being outside the satisfactory range. The remaining samples were acceptable according to criteria proposed by Ambrose and Norr [64] and van Klinken [66]. Fig 3 shows the plot of δ^13^C versus δ^15^N isotope values for both faunal and human samples.

The δ^13^C values of the three herbivore samples (*Cervus elaphus* and *Equus asinuus*) ranged from -21.4‰ to -21.1‰ (mean -21.3 ± 0.1‰), while δ^15^N values ranged between 3.5‰ and 5.9‰ (mean 4.5 ± 1.3‰). Omnivores (*Sus domesticus* and *Hystrix cristata*,) possessed values from 21.8‰ to -20.6‰ (mean -21.0 ± 0.7‰) for δ^13^C and from 3.6‰ to 4.7‰ (mean 4.1 ± 0.6‰) for δ^15^N. The carnivore (*Felix catus*, *Canis familiaris* and unidentified carnivores) data ranged from -20.8‰ to -19.5‰ (mean -20.1 ± 0.46‰) and from 7.5‰ to 8.5‰ (mean 7.8 ± 0.4‰) for δ^15^N. Isotope data for all animals are in a range typically associated with the consumption of C_3_ plants. In terms of nitrogen, herbivores have δ^15^N values within the range found for primary consumers in the Mediterranean area [79]. Omnivore δ^15^N values are similar to those of the herbivores, which indicates that at Allumiere they consumed similar dietary resources, i.e. mainly plants with little/no animal protein. The *Hystrix cristata* (porcupine) show isotope values that are of an essentially herbivorous and it seems that any occasional consumption of insects and small vertebrates [80] did not play a key role in the stable isotope values for this animal. The analysis of the pig diet is useful to understand the variation in husbandry practices [81–88] among the different communities. Indeed, pigs raised in a home-based system are expected to have a more controlled diet than free-range animals. For Medieval northwestern Europe communities, pigs had a diet based on terrestrial plant and human refuse [87], whereas our data show a comparable δ^15^N values between the *Sus domesticus* and the other herbivores analyzed. During the Middle Ages, in some areas (including Lazio) pigs were free to roam in the fallow land close to the city [82], where they would typically consume acorns (pannaga) [89]. Our results seem to suggest that the Allumiere pigs were predominantly fed with plant products, which might indicate that they wandered about in forested environments. Therefore, the difference between Allumiere and data from published northwestern European populations [87] is probably related to the different social status of the communities: the Allumiere population was made up of workers of low social status, while the other data derives from predominantly coastal and elite/urban communities with different husbandry practices.

The human remains from *La Bianca* (Allumiere) possessed δ^13^C values between -20.6‰ and -17.1‰ (mean -19.2 ± 1‰) and δ^15^N values between 5.9‰ and 12.1‰ (mean 8.6 ± 1.4‰). Both nitrogen and carbon stable isotope values for humans showed a high variability, in particular two individuals (SU 245 and 317) have elevated δ^15^N values of 12.1‰ and 11.9‰ and one (SU 388) has a particularly low δ^15^N value of 5.9‰. Despite the variability in isotopic values, no statistically significant difference was observed, between different sexes or age at death. The majority of humans demonstrate an enrichment of ^15^N (about 3–5‰, with a nitrogen offset equal 3.5‰) in comparison to the animals (Fig 3), reflecting a typical trophic level shift indicative of the consumption of animal protein. Most of the individuals have diets with low input of animal protein: the wide range in δ^15^N values for the population (6.2‰) indicates that animal protein made a greater or lesser contribution to the diet of particular individuals. On average, the human-animal offset in is 3.5‰, which is at the lower end of the accepted 3–5‰ between trophic levels [90] and spacing of up to 6‰ has been suggested for humans [91]. Therefore, for many individuals, it seems animal protein did not contribute a major part of the diet, which is in keeping with the low status nature of the population. However, for those possessing δ^15^N values of upwards of 9–10‰, animal protein will have had a major input and for the most elevated values, fish may also have made a contribution. High δ^13^C values (> 18‰) for some individuals (e.g. SU 362, 182, 192 and 319) indicate the consumption of C_4_ plants or marine fish consumption. However, the fact that these individuals also tend to have amongst the lowest δ^15^N values would suggest that the former is most likely.

**Fig 4:**
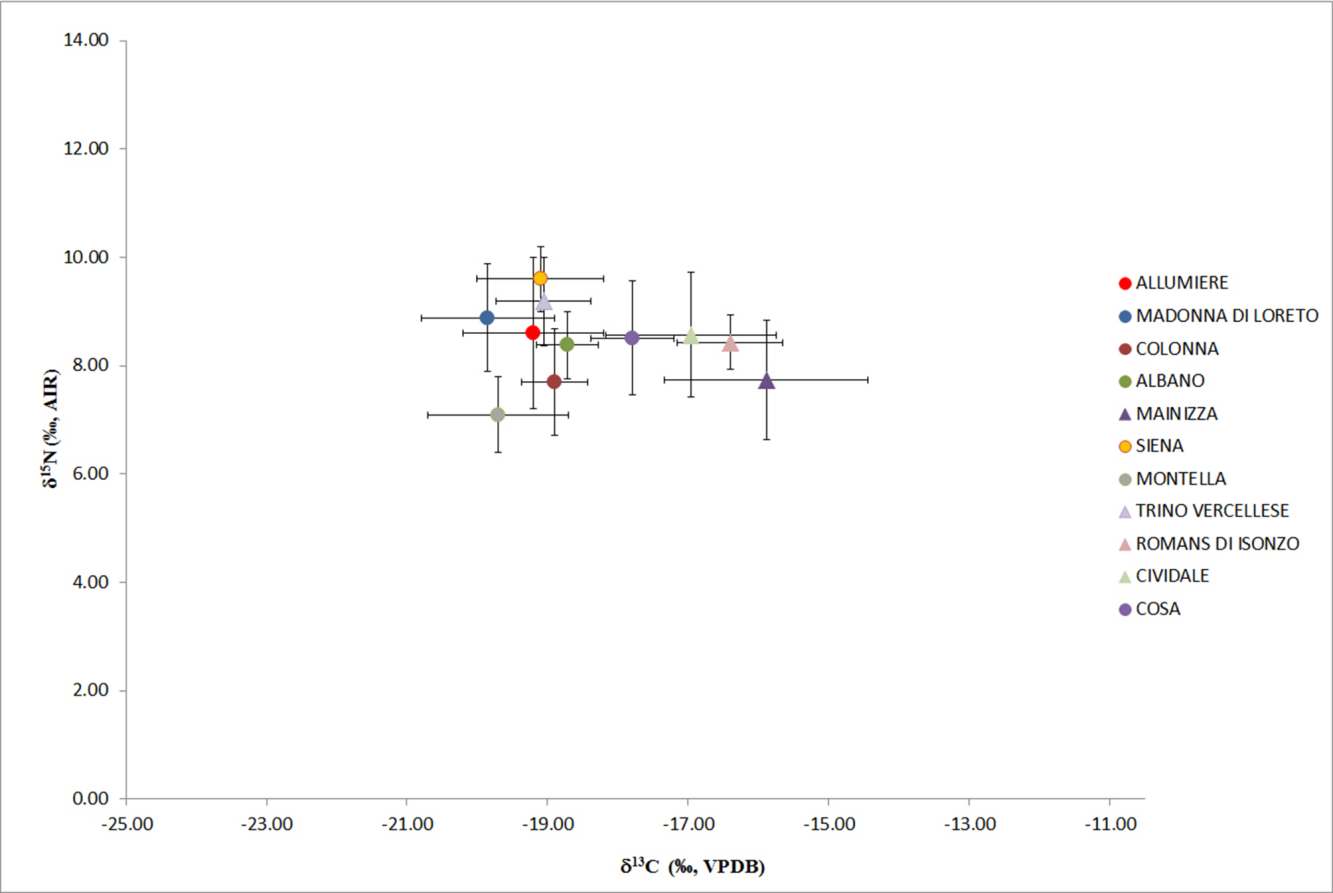
Plot of carbon *vs* nitrogen mean isotopic values and error bars represent ±1σ for human remains from Allumiere and the other Medieval Italian sites. Triangles represent the north Italian populations and circles the central Italian populations. Trino Vercellese (Piedmont) [92]; Pava Piene Siena (Tuscany) [93]; Piazza Madonna di Loreto (Rome) [94]; Cividale, Romans d'Isonzo and Mainizza (Friuli-Venezia Giulia) [95]; Cosa Grosseto (Tuscany) [96]; Albano Laziale Rome (Lazio) [97]; Montella Avellino (Campania) [98]; Colonna Rome (Lazio) [99].

Comparisons of Allumiere with other Medieval published data [92–99] were performed (Fig 4). Significant statistical differences (Kruskal-Wallis test p<0.05), were found between δ^13^C values, mostly with northeastern Italian sites (Cividale, Mainizza, Romans d’Isonzo, Siena), Cosa from entral Italy and Montella (AV) from Campania in southern Italy. For northeastern and central Italy sites, the consumption of C_4_ or marine resources were interpreted to be responsible for enriched δ^13^C values at these sites [95–96]. As regards to Montella [96] the sample was from a Franciscan friary so it is possible to hypothesize that they followed a different dietary plan. Statistically significant differences (Krustal-Wallis test p<0.05) were found between δ^15^N with the sites of Siena and Montella. However, only the site of Siena [93] showed higher mean δ^15^N values, although this could also be due to the small sample size (N = 19). These differences describe a heterogeneous dietary landscape of the Medieval Italian communities probably related to geographical position and chronology.

Analysis of aDNA from dental calculus was carried out to attempt to determine which animals were relied upon and to identify the possibility of fish consumption. Successful results were achieved for 19 individuals out of a total of 35 (Table 4).

**Table 4:** Ancient DNA analysis on dental calculus.

SU	Sheep	Cattle	Chicken	Pig	Fish
319	+	+	-	-	+
303	+	+	-	-	+
245	+	+	-	-	+
318	+	-	-	-	-
204	+	+	+	-	-
330	+	-	-	-	**-**
198	+	+	-	-	**-**
185	+	+	-	+	+
320	+	+	-	-	+
346	+	-	-	-	+
192	+	+	-	-	**-**
176	+	+	+	-	+
290	+	+	-	-	+
169	+	+	+	-	+
272	+	+	-	-	+
159	+	+	-	-	**-**
221	+	+	-	-	**-**
147	**+**	**+**	**-**	**+**	**-**
213	+	+	-	-	**-**
**Total**	**19**	**16**	**3**	**2**	**10**
**%**	**100**	**84.2**	**15.8**	**10.5**	**53**

**Fig 5:**
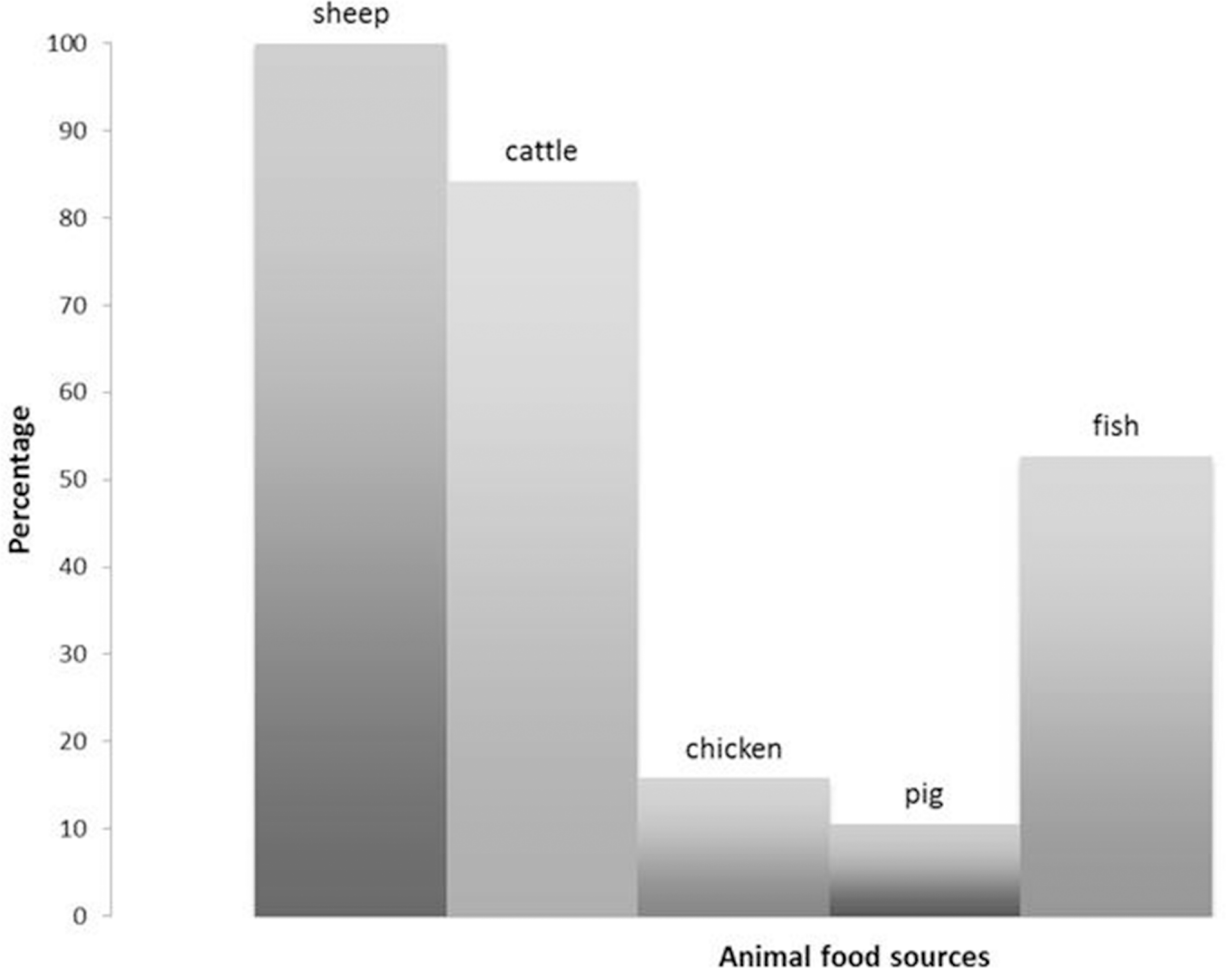
Percentages of consumption of the different animal dietary resources.

In accordance with literature data from different Medieval sites [100–102], among the *La Bianca* community the majority of individuals seemed to consume sheep and cattle products with a minimal amount of fish, pig and chicken (Fig 5).

The use of the DNA analysis from the dental calculus was useful not only to confirm the stable isotope results but also to understand which of the animal species were predominantly consumed.

### Light microscopy analysis

The presence of microfossils in dental calculus was detected in 66% of the individuals. In Table 5, the number of starch granules, pollen grains and other microremains (i.e. phytoliths, calcium oxalate crystals) detected in each sample (lines) was reported, after their taxonomic classification (columns).

**Table 5:** Amount of starch granules (per taxonomic group or total) and other microremains detected in dental calculus. The numbers reflect the quantity of each microremain counted by direct microscopy observation in the samples.

SU	I	II	III	IV	V	VI	VII	VIII	IX	UN	TS	PO	PG
**110**						1		1		1	**3**		
**139**	1				1		1	1			**4**	**2 P+ 1 OCa**	
**147**				3			2	1	1		**7**		
**158**			4		1						**5**	**1 OCa**	
**159**											**0**		
**169**	1					3			1	1	**6**	**1 P**	
**173**											**0**		
**176**									1		**1**		
**185**								1	1		**2**		**1 A**
**192**									1	2	**3**		
**198**		1	24			1	194				**220**		
**204**								1	1		**2**		
**213**											**0**		
**221**									1		**1**		
**239**								1			**1**	**3 P**	
**245**										1	**1**	**1 P**	
**256**											**0**		
**269**										1	**1**		
**272**											**0**		
**290**				1	1					1	**3**		
**303**											**0**		
**304**	1										**1**		
**307**									1	1	**2**	**1 P**	
**308**											**0**	**1 OCa**	
**317**							1				**1**	**1 OCa**	
**318**											**0**	**1 OCa**	
**319**									2		**2**		
**320 Tb.47**			2						1		**3**		
**325**							1				**1**		
**330**											**0**		
**333**						1				1	**2**		**2 L**
**334 Tb.51**		1		2						2	**5**	**1 OCa + 1C**	**1 Nd**
**343 Tb.53**											**0**		
**346 Tb.54**											**0**		
**356**											**0**		
**TOTAL**	**3**	**2**	**30**	**6**	**3**	**6**	**199**	**6**	**11**	**11**	**277**		**4**

Stratigraphic unit (SU); Morphotype I, *Avena* sp. (I); Morphotype II, *Hordeum* sp.(II); Morphotype III, *Myristica fragrans* (III); Morphotype IV, *Piper nigrum* (IV); Morphotype V, *Quercus ilex* (V); Morphotype VI, *Sorghum bicolor* (VI); Morphotype VII, *Triticum* sp. (VII); Morphotype VIII, Fabaceae not determined (VIII); Morphotype IX, Fagaceae not determined (IX); Unidentified starch granules (UN); Total of starches (TS); Phytoliths and other microremain (PO); Pollen grains (PG); Poaceae phytoliths (P); calcium oxalate crystals (OCa); Cucurbitaceae fruit epicarp fragment (C); Asteraceae pollen grain (A); *Laurus nobilis* pollen granules (L); not determined pollen grain (Nd).

The 277 starch granules detected by LM were clustered in 9 different morphotypes, on the basis of morphometric and morphological parameters (Table 6).

**Table 6:** Starch granule morphotypes recovered from the dental calculus of the studied individuals.

Morphotype	Taxonomic group	Morphologic and morphometric description
**I**	*Avena* sp.	Multifaceted polyhedral shape on one side and dome shaped on the other one; individual granule size: 3–8 μm in length and in width.
**II**	*Hordeum sp*.	Granules were rounded or disk shaped; size range: 4–19 μm in length and 3–15 μm in width; a centric *hilum* is distinct; close concentric *lamellae* were more detectable in the central area. One of them was attributable to the *H*. *vulgare* species as it showed longitudinal fissure located on lateral peripheral margin
**III**	*Myristica fragrans*	Granules were compound, essentially dimers or trimers. The single subunit is rounded with some peculiar flattened surfaces; size range: 5–9 μm in length and 4–5 μm in width; multiple fissures radiate from the centric *hilum*; *lamellae* are not detectable.
**IV**	*Piper nigrum*	Polyhedral granules with pentagonal or hexagonal concave faces and acute edges; size range: 1–2 μm both in length and in width; typical bright boundary.
**V**	*Quercus ilex*	Grains were drop-shaped; size range: 5–8 μm in length and 4–6 μm in width; *hilum* not clearly evident; presence of the typical hole at the narrow end.
**VI**	*Sorghum bicolor*	Rounded granules with some peculiar flattened surfaces; size range: 2–15 μm in length and 2–10 μm in width; deep radial fissures starting from a centric *hilum*. *Lamellae* were not detectable.
**VII**	*Triticum* sp.	Granules were disc-shaped; size range: 6–21 μm in length and 4–18 μm in width; *hilum* not visible; weakly concentric *lamellae* were present. Three granules were attributable to the *T*. *dicoccum* species as they showed clear concentric kidney-shaped *lamellae* distributed on the whole granule.
**VIII**	Fabaceae Nd	Irregular reniform granules; size range: 6–19 μm in length and 5–14 μm in width; *hilum* is not detectable; quite clear concentric *lamellae*; presence of a longitudinal crack in the amorphous central area.
**IX**	Fagaceae Nd	Oblong granules; size range: 5–15 μm in length and 3–11 μm in width; faintly visible *lamellae*; presence of longitudinal fissure.

Moreover, unidentified remains showing no distinctive features were also recorded. The highest percentage of individuals presented starch of morphotype IX (Fagaceae), followed by miners who consumed morphotype I, II, VI and VII (Poaceae). Particular attention was paid to SU 198 individual, whose dental calculus presented an aggregate containing 220 starch granules, essentially ascribable to morphotype VII (*Triticum* sp., 192 granules) and to morphotype III (*Myristica fragrans* Houtt., 24 granules).

**Fig 6:**
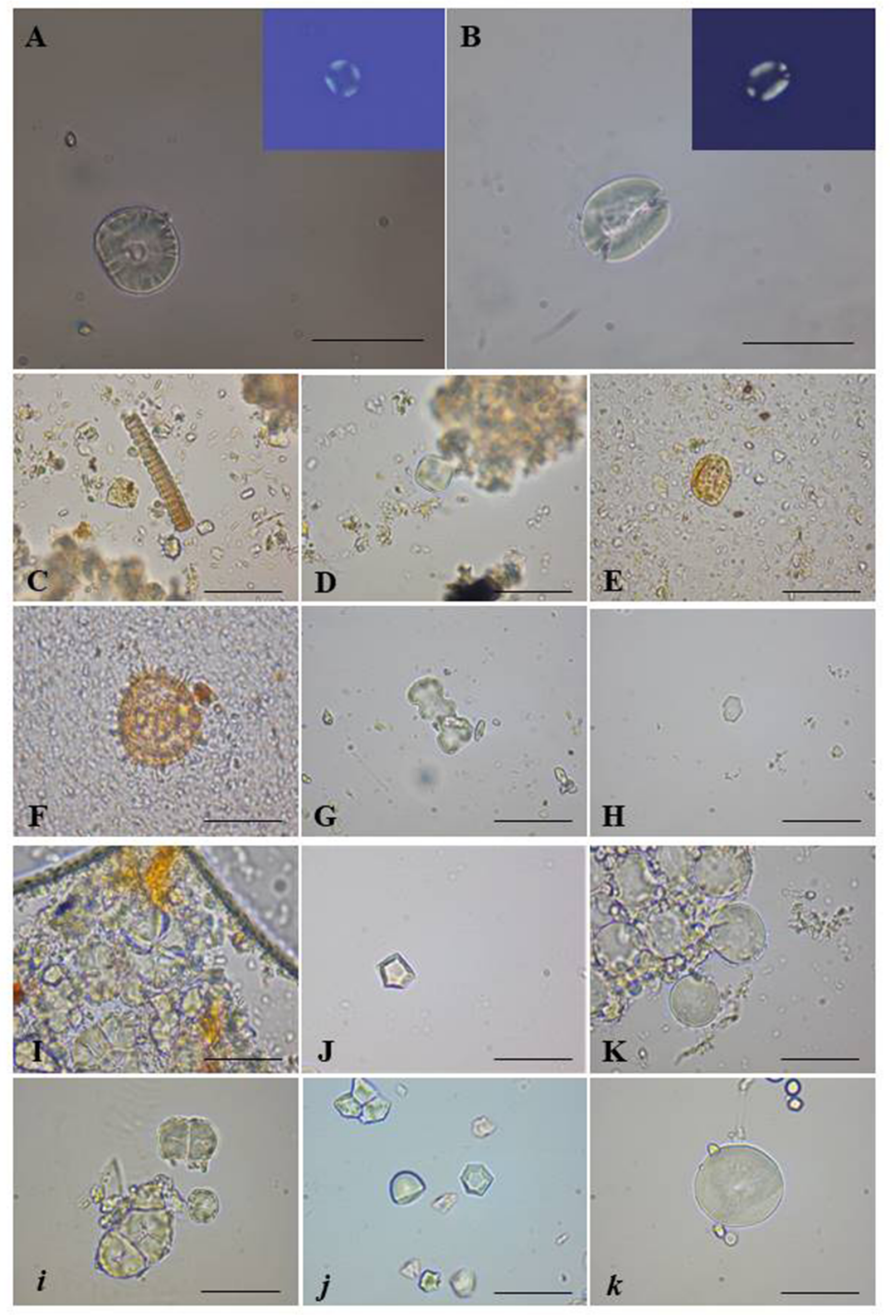
Examples of plant microremains found in the dental calculus of the samples. Starch granule of *Triticum* sp. and relative polarized image (A); starch granule of Fabaceae and relative polarized image (B); Poaceae phytoliths (C); not determined starch granule (D); fragment of Cucurbitaceae fruit epicarp (E); pollen grain of Asteraceae (F); Poaceae phytoliths (G); calcium oxalate crystal (H); starch granules of *Myristica fragrans* (I) and relative modern experimental reference (i); starch granule of *Avena* sp. (J) and modern experimental reference of *Avena fatua* (j); starch granules of *Triticum* sp. (K) and modern experimental reference of *Triticum durum* (k). The black bar indicates 15 μm.

Beyond starch granules, microscopic analysis also revealed, the presence of pollen grains of Asteraceae and *Laurus nobilis* L. in the dental calculus of the individuals. In addition, 8 phytoliths belonging to Poaceae family, 6 calcium oxalates crystals and a Cucurbitaceae fruit epicarp were observed. Examples of images of microremains found in calculus samples are indicated in Fig 6.

### GC-MS analysis

GC-MS analysis produced results for 23 individuals. The analysis was repeated for each sample at least 3 times and, in all cases, it always showed similar profiles. In S6 Table the chemical compounds identified in the calculus by GC-MS are listed, clustered by biochemical class. For each chromatographic profile, specific foods (i.e. *Artemisia dracunculus* L.) or food categories (i.e. milk and derivatives, plant oils), consumed in life at least once by the community of *La Bianca* were extrapolated, associating the detected compounds as reported in literature and scientific food databases [103–104]. Generally, markers of Brassicaceae (i.e. desulfoglucobrassicin; isothiocyanatoacetaldehyde dimethyl acetal; isothiocyanic acid, propyl ester; 13-docosenoic acid, methyl ester) and milk and its derivatives (i.e. beta-lactose) were the most recurrent molecules in the dental calculus of the population, followed by plant oils (i.e. oleic acid; 8,11,14-eicosatrienoic acid, methyl ester, (Z,Z,Z); 9,12-octadecadienoic acid, methyl ester, (E,E)) and theophylline, an alkaloid produced in leaves of *Camellia sinensis* (L.) Kuntze. The detection of other plant chemical compounds (i.e. lactones, terpens) suggested the key role of plants (i.e. spices, herbs, fruits of Rosaceae) in the miners’ diet. Moreover, evidence of pharmacologically active alkaloids (i.e. stachydrine; securinine; conhydrin) supported the knowledge and application of medicinal plants (i.e. *Ephedra* sp., *Stachys officinalis* (L.) Trevis., *Artemisia dracunculus*, *Conium maculatum* L. and *Securinega suffruticosa* (Pall.) Rehd).

## Discussion

Archaeological evidence suggests that the archaeological site of *La Bianca* (Allumiere) is relative in date to one of the first recorded human settlements in the area aimed to alum production. Musculoskeletal stress markers, combined with degenerative disease patterns provided important results in the reconstruction of miners’ working activity. Morphological changes depend on repeated daily exercises that stimulate bone remodeling at the attachment sites, increasing blood flow as a consequence [105–106]. However, although the macroscopic analysis of bone morphological modifications can be related to muscles subjected to a high biomechanical stress, often the absence of archaeological data or documents confirming the validity of these assumptions does not allow determining the exact activity carried out in the individual’s lifetime [107].

The present work represents a unique case study, providing evidence of a relationship between occupational stress markers of bone tissue and working activities [78]. This oddity is partly due to the accurate and detailed description of the four phases of alum production that is available from historical written sources [1–3]. The excavation procedure represented the most physically taxing phase, where picks were used to extract alunite from the rocks. This activity may have also involved climbing with narrow strings on the chest. Calcination was another complex phase, where alunite stones were roasted for 12–14 hours at 600–700°C. At the end of this process, alunite was cooled through a generous watering, in order to break the “roasted” stones and obtain a doughy solution: this phase, defined *moistening*, could last up to 60 days. The last step was the lixiviation during which the moistened solution was purified by mixing for 24 hours. Even if all four phases (i.e. excavation, calcination, moistening and lixiviation) markedly involved the shoulder girdle, the different scheme of enthesal changes and osteoarthritis pattern observed on the specimens seemed to differ in relation to the biomechanical stress caused by the different tasks; thus allowing the classification of the individuals in four main clusters, each one corresponding to the working phase they were probably most involved in. It is known that osteoarthritis may be a result of traumas or infections, certainly the biomechanical stress suffered from the joint represents one of its causative factors [108] even if the aetiology is far from being fully understood [109]. These data were strongly supported by osteometrics, which reported the existence of different strength and asymmetry patterns among individuals. A tall and robust skeleton could be attributable to a probable selection of individuals to be miners in relation to their adaptability to such type of work. Working tasks and related biomechanical stress seemed to have influenced also the prevalence of skeletal traumas and pathologies. In particular, a comparison with the Latium Medieval populations of Colonna [99] and Santa Severa [110] showed significant differences in the observed pathological pattern. In Colonna and Santa Severa, periostitis was detected in 4.2% and 4.4% of the individuals, respectively, *vs* the value of 32% found in Allumiere. Similarly, degenerative diseases affected only 16.7% and 22.3% of the skeletal remains, in the two coeval cases, compared to 86% of *La Bianca* community. The morphological examination demonstrated peculiar and unique features on the analyzed sample showing that it is also one of the few Medieval examples that allows a clear identification of the social status of its components. The combined analysis of enthesal changes and osteoarthritis led to interesting results however the exact relationship between morphological variations on bone tissue and the activity carried out should be considered as a plausible hypothesis. It is known that age at death could also play an important role in the morphology of enthesal changes and that the different studies in literature underline the difficulties in finding a real correlation between entheses and activity patterns [111–113].

Stable isotope data supported a diet based on terrestrial proteins. The δ^13^C values also suggested a contribution of C_4_ plants to the diet, as confirmed by archaeobotanical microscopy evidence. Indeed, the use of C_4_ plants as food, such as millet, has been documented in Italy since the Bronze Age [114], and continued in the Medieval period at least in the north east of Italy [95].

DNA analysis revealed the consumption of animal proteins deriving from sheep and cattle meat, whereas pig and chicken use seemed to be negligible. Historical evidence indicates that the consumption of chicken remained low in the Middle Ages, corroborating our results [115]. Similarly, as we observed in the Medieval period, the use of pork meat never reached the elevated consumption of the first century of the Roman Imperial Age [79].

The dietary evidence presented by GC-MS and starch analysis will not be quantifiable in relation to the amounts consumed, however they do give an indication of the types of foods ingested during life, particularly when considered at the population level [20]. The microscopic analysis showed a consumption of Fagaceae, followed by C_3_ Poaceae caryopses (i.e. *Hordeum* sp., *Triticum* sp.). Acorns, being rich in proteins, unsaturated fat, carbohydrates, minerals and vitamins, represented a food with high energy value [116–118]. Indeed, they were commonly added to other cereal flours (after elimination of indigestive tannins by thermal treatment in water) or prepared as anti-diarrheal and astringent decoctions [119]. The high number of starch granules that were unable to be taxonomically identified could be due to modifications induced by cooking processes, ptialin enzymimatic activity or grinding procedures. Among the miners, individual SU 198 in particular presented a higher number of starch granules of *Triticum* sp. and *Myristica fragrans* (nutmeg). This Asian spice was also detected in the dental calculus of other two individuals, suggesting the use of this plant species in the studied community. Nutmeg trades have been documented in the Mediterranean area since the 6^th^ century, although archaeological findings are rare. It was usually grounded and mixed with red wine or used, in medicine, for its anti-inflammatory properties [120–121]. GC-MS analysis revealed that more than half of the individuals consumed Brassicaceae and dairy products, confirming the fundamental role of these foodstuffs in the Medieval diet. At the population level, the consumption of plant oils, contained for example in *Olea europaea* fruits or acorns [119, 122–123], was observed. In addition, the detection of theophylline, an alkaloid with diuretic power, suggested the use of plant species native of South-East Asia, such as *Camellia sinensis* (the tea plant). To a lesser extent, the use of foods containing cholesterol (perhaps meat, cheese and eggs), *Artemisia dracunculus* (tarragon, spice with antiseptic, anti-inflammatory and digestive properties) [124], Rosaceae fruits (i.e. lactones) and various herbs were also detected. The identification of secondary metabolites, essentially alkaloids, with pharmaceutical properties suggested the knowledge of medicinal plant species, such as *Stachys officinalis* (astringent, digestive and sedative) [125] and *Ephedra* sp. (bronchodilator) [126]. The detection of a metabolite typical of Cucurbitaceae (cucumber aldehyde) supported the use of species such as cucumber and squash; this evidence was also confirmed by the presence, in the calculus of a specimen, of a fragment of cucumber fruit epicarp [127].

Individual SU 176 indicated the consumption of *Conium maculatum* (hemlock) known to be poisonous but used in low doses in the Middle Ages, along with extract of *S*. *officinalis* and fennel seeds, as remedy against epilepsy, muscle spasms, impotence, tubercular adenitis and bites from dogs infected with rabies [128]. Individual SU 185 had evidence for *Sorbus domestica*, a small tree of the Rosaceae family used in the past for its fruits, called sorbs, rich in vitamin C and individual SU 110 showed evidence for *Securinega suffruticosa*, an oriental plant containing secondary metabolites that act by stimulating the central nervous system [129]. The peculiar use of the *S*. *suffruticosa*, *C*. *sinensis* and *M*. *fragrans*, typical of Asian areas, could be explained by the existence of commercial trades with Eastern regions or the presence, among the miners, of individuals with Eastern origin, which probably introduced these medicinal species in the community. This latter hypothesis is in line with archaeological and historical data on alum production, a process widely developed in Turkey and more in general in the Eastern Europe [1–3]. It is not possible to exclude the idea that, in order to establish the first Italian alum extraction system, the Pontifical State would have called on foreign experts. This hypothesis is corroborated by the presence of four individuals with shovel teeth, a typical feature of Eastern origin individuals [130].

This present combined approach allowed us to obtain a more detailed appreciation of the diet. In particular, two individuals SU 317 and SU 245 showed an enrichment of δ^15^N values >3.5‰ than the maximum animal measurements which indicated the consumption of protein enriched in ^15^N, perhaps from aquatic sources [131–132], but note that trophic level enrichment for humans has been reported up to 6‰ [133]. Analysis of DNA from dental calculus, performed only on SU 245, suggested marine fish consumption. We hypothesized that this enrichment of δ^15^N value could be also associated to breastfeeding in SU 317 (which was a juvenile individual aged between 2–3 years old) [134] and possibly to nutritional stress in SU 245, who suffered from a compound fracture on the tibia and fibula (Fig 2). It is widely documented that food deficiencies activate gluconeogenesis, a biochemical process, which uses non-carbohydrate sources to produce glucose [96, 135–137], and increases δ^15^N values in tissues [138]. Furthermore, GC-MS analysis of the dental calculus of SU 245 revealed the presence of markers of *A*. *dracunculus*, a plant documented to be an anesthetic and anti-inflammatory [124]. It is reasonable to believe that a compound fracture, as that detected in SU 245, caused pain and induced the application of drugs and medical treatments to treat this critical condition.

In conclusion, applying an innovative and original multidisciplinary approach, we present a detailed osteobiography of the first Italian community of alum miners. In detail, according to morphological features, we hypothesized the working task of each individual and reconstructed their dietary patterns. Furthermore, archeobotanical analysis demonstrated that miners used spices and herbs endemic of Asia, for therapeutic purposes, revealing their knowledge about Eastern traditional medicine.

## Supporting information

S1 Table List of the skeletal remains analyzed in the present research housed at the Department of Biology of the University of Rome “Tor Vergata”. (DOCX)

S2 Table Primer sequences used to amplify animal different DNA regions, size of the PCR product obtained and annealing temperature. (DOCX)

S3 Table Results of the post-cranial indices. (nr: not recordable). (DOCX)

S4 Table Presence of pathology/stress markers for each individual. Neoplasia (NP), Infectious diseases (periostitis, PO; osteomyelitis, OM; infections, IN), trauma and stress markers (fractures, FR; grasping GR), degenerative pathologies (degenerative diseases, DG; and axial degereative diseases (Schmörl’s nodes, SN)) and congenital disorders (CO), and inflammation (IF).
(DOCX)

S5 Table Results of the analysis on degenerative disease pattern (nr: not recordable). (DOCX)

S6 Table The molecules identified in dental calculus by GC-MS analysis were listed and clustered in biochemical classes for each sample. (DOCX)
