# Supplementary material for: Who were the miners of Allumiere? A multidisciplinary approach to reconstruct the osteobiography of an Italian worker community
Source: PLoS One. 2018 Oct 11;13(10):e0205362. doi: 10.1371/journal.pone.0205362 (PMC6181348; doi:10.1371/journal.pone.0205362)
Supplement: S1 Table — (DOCX) [file pone.0205362.s001.docx]

**S1 Table. List of the skeletal remains analyzed in the present research housed at the Department of Biology of the University of Rome “Tor Vergata”.**

| **Box Number** | **Skeletal remains / Stratigraphic Unit (SU)** |
| --- | --- |
| 1 | 280 |
| 2 | 290 |
| 3 | 231 |
| 4 | 270 |
| 5 | 274 |
| 6 | 213 |
| 7 | 296 |
| 8 | 245 |
| 9 | 101 C A*/168 C A*/101 A* 385 A*/399.2/399 A*/101.3 A* |
| 10 | 339 |
| 11 | 176 |
| 12 | 239 |
| 13 | 147 |
| 14 | 139 |
| 15 | 234 |
| 16 | 237 |
| 17 | 269 |
| 18 | 277 |
| 19 | 288 |
| 20 | 283 |
| 21 | 374 A*/355 A*/385.2 A* 101.4 A*/101.2 A* |
| 22 | 249 |
| 23 | 256 |
| 24 | 110 |
| 25 | 226 |
| 26 | 293 |
| 27 | 201 |
| 28 | 159 |
| 29 | 356 |
| 30 | 135 |
| 31 | 299 |
| 32 | 192 |
| 33 | 388 |
| 34 | 198 |
| 35 | 359 |
| 36 | 204 |
| 37 | 216 |
| 38 | 385.3/385.5/385.6 |
| 39 | 207 |
| 40 | 362 |
| 41 | 330 |
| 42 | 346 |
| 43 | 346 |
| 44 | 334 |
| 45 | 343 |
| 46 | 144 |
| 47 | 307 |
| 48 | 382 |
| 49 | 308 |
| 50 | 311 |
| 51 | 325 |
| 52 | 318 |
| 53 | 319 |
| 54 | 284 |
| 55 | 303 |
| 56 | 304 |
| 57 | 381 |
| 58 | 317/324 |
| 59 | 173 |
| 60 | 169 |
| 61 | 182 |
| 62 | 179 |
| 63 | 221 |
| 64 | 320 |
| 65 | 189 |
| 66 | 195 |
| 67 | 190 |
| 68 | 185 |
| 69 | 158 |
| 70 | 416 |
| 71 | 383 |
| 72 | 238 |
| 73 | 210 |
| 74 | 272 |
