# Supplementary material for: Who were the miners of Allumiere? A multidisciplinary approach to reconstruct the osteobiography of an Italian worker community
Source: PLoS One. 2018 Oct 11;13(10):e0205362. doi: 10.1371/journal.pone.0205362 (PMC6181348; doi:10.1371/journal.pone.0205362)
Supplement: S2 Table — (DOCX) [file pone.0205362.s002.docx]

**S2 Table.** Primer sequences used to amplify animal different DNA regions, size of the PCR product obtained and annealing temperature.

| **Primer** | **Species** | **Sequence (5’-3’)** | **Size of PCR product**  **(bp)** | **T annealing** |
| --- | --- | --- | --- | --- |
| Bov84-F | cattle | TTAGTTGAATTAGGCCATGAAGCA | 84 | 54 °C |
| Bov84-R | cattle | GTTTAAATAGGGTTAAGATGCACTCAATC |  |  |
| Sus85-F | pig | GGAGCAGTGTTCGCCATTATA | 85 | 57 °C |
| Sus85-R | pig | TTTTTGCTCATGCTTGGTTG |  |  |
| Ovis-F | sheep | CCCTAGGTTTCATCTTTCTTTTCACA | 67 | 60 °C |
| Ovis-R | sheep | AGGGAGGAGTTGGCTAGAACAA |  |  |
| Gall-F | chicken | AACCTCCTCCAGCGGATAATAAT | 66 | 60 °C |
| Gall-R | chicken | TTTGTTGGTGGCTGCTTGAA |  |  |
| miniA_F | fish | CACGACGTTGTAAAACGACACIAAICAIAAAGAYATYGGC | 129 | 46 °C |
| miniA_R | fish | GGATAACAATTTCACACAGGAARAAAATYATAACRAAIGCRTGIGC |  |  |
| miniC_F | fish | CACGACGTTGTAAAACGACACYAAICAYAAAGAYATIGGCAC | 127 |  |
| miniC_R | fish | GGATAACAATTTCACACAGGGAARATCATAATGAAGGCATGIGC |  |  |
