# Supplementary material for: Who were the miners of Allumiere? A multidisciplinary approach to reconstruct the osteobiography of an Italian worker community
Source: PLoS One. 2018 Oct 11;13(10):e0205362. doi: 10.1371/journal.pone.0205362 (PMC6181348; doi:10.1371/journal.pone.0205362)
Supplement: S3 Table — (DOCX) [file pone.0205362.s003.docx]

**S3 Table. Results of the post-cranial indices. (nr: not recordable; R: right, L: left)**

| **METRIC MARKERS OF FUNCTIONAL/NUTRITIONAL STRESS** | | | | | | | | | | | | | | | | | | | | |
| --- | --- | --- | --- | --- | --- | --- | --- | --- | --- | --- | --- | --- | --- | --- | --- | --- | --- | --- | --- | --- |
| **SU** | **HUMERUS** | | | **ULNA** | | | | **RADIUS** | | | **FEMUR** | | | | **TIBIA** | | | | **CLAVICLE** | |
|  | **DIAPHYSEAL INDEX** | | | **OLENIC INDEX** | | | | **DIAPHYSEAL INDEX** | | | **PILASTRIC INDEX** | | **PLATYMERIC INDEX** | | **DIAPHYSEAL INDEX** | | **CNEMIC INDEX** | | **DIAPHYSEAL INDEX** | |
|  | **R** | **L** | | **R** | | **L** | | **R** | **L** | | **R** | **L** | **R** | **L** | **R** | **L** | **R** | **L** | **R** | **L** |
| 110 | nr | 91.3 | | nr | | 92.0 | | nr | 62.9 | | nr | nr | nr | nr | nr | nr | nr | nr | 136.4 | 145.5 |
| 135 | nr | nr | | 78.8 | | nr | | nr | nr | | nr | nr | nr | nr | nr | nr | nr | nr | nr | nr |
| 139 | nr | nr | | nr | | nr | | nr | nr | | nr | 115.0 | nr | 78.7 | nr | 72.6 | nr | 71.3 | 145.9 | nr |
| 144 | 83.3 | nr | | 91.7 | | 74.9 | | 72.2 | 66.7 | | nr | nr | nr | 84.9 | nr | nr | nr | nr | 90.9 | nr |
| 147 | 90.9 | nr | | 84.6 | | 81.5 | | 71.4 | 63.9 | | nr | 110.0 | 88.7 | nr | nr | nr | nr | nr | nr | 80.8 |
| 158 | 89.5 | 83.3 | | 96.0 | | 92.0 | | nr | 75.0 | | 103.3 | 119.0 | 96.3 | nr | nr | nr | nr | nr | nr | nr |
| 169 | nr | nr | | nr | | nr | | nr | nr | | 113.2 | 107.1 | 82.8 | 78.3 | nr | nr | 74.6 | 75.0 | nr | nr |
| 173 | 88.1 | 88.1 | | nr | | nr | | nr | nr | | 102.6 | 93.1 | 73.0 | 67.0 | nr | nr | nr | 66.1 | nr | nr |
| 176 | 73.9 | 81.0 | | 83.0 | | nr | | 76.0 | 81.9 | | 109.1 | nr | nr | nr | nr | nr | 81.2 | nr | nr | nr |
| 185 | 85.2 | 79.8 | | 97.7 | | 87.0 | | nr | nr | | nr | 102.3 | nr | 78.5 | nr | nr | nr | nr | nr | nr |
| 189 | nr | nr | | 91.3 | | 89.6 | | 73.5 | nr | | 111.5 | 113.0 | 88.9 | 95.7 | 78.3 | 75.4 | 71.4 | 73.5 | nr | 100.0 |
| 192 | nr | 80.2 | | nr | | nr | | nr | nr | | nr | 105.8 | nr | 70.2 | 79.8 | nr | 71.3 | nr | 122.9 | 112.0 |
| 195 | nr | 77.2 | | nr | | 80.0 | | nr | 64.7 | | nr | nr | nr | nr | nr | nr | nr | nr | nr | 100.0 |
| 198 | nr | 83.3 | | nr | | nr | | nr | 72.2 | | nr | nr | nr | nr | nr | nr | nr | 77.8 | nr | 140.0 |
| 201 | nr | 84.4 | | 93.6 | | 89.9 | | 81.7 | 75.4 | | nr | nr | nr | nr | 81.5 | nr | 80.0 | nr | nr | nr |
| 204 | 86.3 | 78.4 | | 93.5 | | 88.2 | | 75.8 | nr | | 112.1 | 103.7 | 95.6 | 88.2 | 79.4 | 81.3 | 78.4 | 71.9 | 155.3 | 137.5 |
| 221 | 90.9 | 93.8 | | 69.1 | | 63.5 | | 72.7 | 65.8 | | nr | 103.4 | nr | 67.0 | nr | 73.2 | nr | 74.0 | 113.6 | 100.0 |
| 231 | nr | nr | | nr | | nr | | 63.2 | 59.0 | | nr | nr | nr | 86.5 | nr | nr | nr | nr | nr | nr |
| 239 | 86.3 | 90.5 | | 75.9 | | 76.3 | | 79.2 | 81.6 | | 100.0 | 96.7 | nr | nr | 75.8 | 80.6 | 76.5 | 79.0 | nr | 81.9 |
| 245 | 82.6 | 81.4 | | 85.6 | | 87.2 | | 73.9 | 73.5 | | 110.2 | 107.1 | 85.4 | 85.2 | 85.8 | nr | 82.0 | nr | 128.6 | 135.0 |
| 249 | nr | nr | | nr | | nr | | nr | 68.6 | | nr | nr | 85.4 | 100.0 | 93.5 | nr | 75.0 | 72.2 | nr | nr |
| 256 | nr | nr | | nr | | nr | | nr | 75.8 | | nr | nr | nr | nr | nr | nr | nr | nr | nr | nr |
| 269 | nr | nr | | 91.3 | | nr | | 75.0 | 74.2 | | 103.6 | nr | 91.8 | 83.6 | 77.4 | nr | nr | 72.2 | nr | 100.0 |
| 272 | 86.9 | nr | | nr | | nr | | nr | nr | | 100.0 | nr | 93.0 | nr | nr | nr | nr | nr | nr | nr |
| 274 | 86.9 | nr | | 83.3 | | 76.0 | | 82.2 | nr | | 114.8 | 109.5 | nr | nr | 87.0 | nr | 79.7 | nr | nr | nr |
| 277 | nr | 83.0 | | 94.8 | | 92.7 | | 67.8 | 74.6 | | nr | 101.7 | nr | nr | 82.9 | 83.2 | 82.9 | 79.3 | nr | nr |
| 280 | nr | nr | | 75.0 | | nr | | nr | nr | | 107.4 | nr | nr | 96.2 | 82.9 | 83.2 | 82.9 | 79.3 | nr | nr |
| 290 | nr | nr | | nr | | nr | | nr | 63.9 | | nr | nr | nr | nr | nr | nr | nr | nr | nr | nr |
| 296 | 87.1 | 89.0 | | 67.5 | | 81.8 | | 64.0 | 55.3 | | 100.0 | 93.1 | 79.5 | 84.8 | 77.7 | 77.2 | 73.8 | nr | nr | nr |
| 303 | 82.1 | 82.7 | | 67.5 | | 81.8 | | 71.8 | 65.5 | | 105.0 | 104.3 | nr | nr | nr | nr | nr | nr | nr | nr |
| 307 | nr | nr | | nr | | nr | | 79.4 | 78.8 | | nr | nr | nr | nr | nr | nr | nr | nr | nr | nr |
| 308 | 88.8 | 85.8 | | 95.2 | | 96.4 | | 82.7 | 85.9 | | 113.8 | 108.3 | nr | nr | 69.7 | 74.9 | 74.0 | 75.9 | 67.4 | 68.8 |
| 311 | 82.7 | 83.7 | | 83.2 | | nr | | 88.9 | 79.2 | | 106.5 | 103.7 | nr | nr | nr | 74.3 | 69.4 | 75.1 | 89.0 | 73.8 |
| 318 | 79.1 | 86.9 | | nr | | 84.6 | | nr | 76.5 | | nr | nr | 82.0 | nr | 78.6 | 72.4 | 80.6 | nr | nr | 116.7 |
| 319 | 79.0 | 78.3 | | 78.2 | | 83.8 | | 73.0 | 73.0 | | 117.9 | 105.0 | nr | nr | 73.5 | 73.5 | 67.2 | 69.2 | 102.6 | nr |
| 320 | nr | nr | | nr | | nr | | nr | nr | | nr | nr | nr | nr | nr | nr | nr | nr | nr | nr |
| 325 | nr | nr | | 86.4 | | 78.3 | | 70.6 | 66.7 | | nr | nr | 84.7 | 71.9 | 85.7 | 78.6 | nr | 74.3 | nr | nr |
| 330 | 75.0 | 74.5 | | 87.8 | | 81.2 | | 73.7 | 70.4 | | 115.4 | 116.1 | nr | nr | nr | 76.7 | 74.8 | 73.3 | 89.7 | 91.9 |
| 343 | 79.1 | nr | | nr | | nr | | 79.4 | nr | | 118.5 | nr | nr | nr | nr | 75.0 | nr | nr | 120.0 | 130.0 |
| 346 | 73.3 | 71.6 | | 79.5 | | nr | | 78.3 | 75.5 | | 121.6 | 116.1 | nr | nr | nr | 76.2 | 66.8 | 68.8 | nr | 76.3 |
| 356 | 84.3 | 77.5 | | 91.0 | | 90.2 | | 83.3 | 86.0 | | 104.9 | 104.9 | nr | nr | 68.8 | 84.2 | 69.2 | 73.1 | nr | 87.0 |
| 362 | nr | nr | | nr | | nr | | nr | nr | | nr | 105.9 | nr | 80.3 | nr | nr | nr | nr | 127.1 | nr |
| **Mean value** | 83.7 | 82.9 | | 84.9 | | 84.0 | | 75.4 | 71.9 | | 109.1 | 106.3 | 86.3 | 82.2 | 79.9 | 77.2 | 75.6 | 73.8 | 114.6 | 104.3 |
| **ROBUSTNESS INDEX** | | | | | | | | | | | | | | | | | | | | |
| **SU** | **HUMERUS** | | | **ULNA** | | | | **RADIUS** | | | **FEMUR** | | **TIBIA** | | **CLAVICLE** | | **FIBULA** | | | |
|  | **R** | **L** | | **R** | | **L** | | **R** | **L** | | **R** | **L** | **R** | **L** | **R** | **L** | **R** | | **L** | |
| 110 | nr | 22.1 | | nr | | 18.7 | | nr | 19.3 | | nr | nr | nr | nr | 31.4 | 32.6 | nr | | nr | |
| 139 | nr | nr | | nr | | nr | | nr | nr | | nr | 13.4 | nr | 21.3 | 28.7 | nr | nr | | nr | |
| 144 | 21.2 | nr | | 19.8 | | nr | | nr | nr | | nr | nr | nr | nr | 25.5 | nr | nr | | nr | |
| 147 | nr | nr | | nr | | nr | | nr | 20.5 | | nr | nr | nr | nr | nr | 26.6 | nr | | nr | |
| 158 | 21.3 | 20.4 | | nr | | 17.5 | | nr | 20.4 | | nr | nr | nr | nr | nr | nr | nr | | nr | |
| 169 | nr | nr | | nr | | nr | | nr | nr | | 12.3 | 12.8 | nr | nr | nr | nr | nr | | nr | |
| 173 | 21.0 | 20.4 | | 17.5 | | nr | | 19.1 | 20.5 | | 15.5 | 11.1 | 26.0 | 23.2 | 29.6 | 26.4 | nr | | nr | |
| 176 | nr | nr | | nr | | nr | | nr | nr | | nr | nr | nr | nr | nr | nr | nr | | nr | |
| 185 | nr | 18.8 | | 15.2 | | 15.1 | | nr | 17.8 | | nr | 12.3 | nr | nr | nr | nr | nr | | nr | |
| 189 | nr | nr | | nr | | 16.7 | | 21.6 | nr | | 13.6 | 14.2 | 24.1 | 24.3 | nr | 27.4 | nr | | nr | |
| 192 | nr | 19.7 | | nr | | nr | | nr | nr | | nr | 12.8 | 26.4 | nr | 30.9 | 29.4 | nr | | nr | |
| 195 | nr | nr | | nr | | 20.1 | | nr | nr | | nr | nr | nr | nr | nr | 27.8 | nr | | nr | |
| 198 | nr | 21.6 | | nr | | nr | | nr | 19.9 | | nr | nr | nr | nr | nr | nr | nr | | nr | |
| 201 | nr | 22.6 | | 20.0 | | 18.4 | | 21.4 | 23.1 | | nr | nr | 21.3 | nr | nr | nr | nr | | nr | |
| 204 | 21.1 | 20.9 | | 20.7 | | 19.9 | | 21.0 | nr | | 13.2 | 12.7 | nr | nr | 27.6 | 26.5 | nr | | nr | |
| 221 | 22.2 | 22.0 | | 19.3 | | 20.3 | | 20.2 | 20.3 | | nr | 12.6 | nr | 20.9 | 24.5 | 23.6 | nr | | nr | |
| 231 | nr | nr | | nr | | nr | | 20.2 | nr | | nr | nr | nr | nr | nr | nr | nr | | nr | |
| 239 | 20.6 | 19.6 | | 18.0 | | 18.2 | | 19.4 | 18.5 | | 13.1 | 12.4 | 21.5 | 20.7 | nr | 26.3 | nr | | nr | |
| 245 | nr | nr | | nr | | 17.5 | | 20.4 | 20.3 | | 12.0 | nr | 20.2 | nr | 31.4 | 30.1 | nr | | nr | |
| 249 | nr | 22.6 | | nr | | nr | | nr | 22.1 | | nr | nr | 22.0 | 22.5 | nr | nr | nr | | nr | |
| 256 | nr | nr | | nr | | nr | | nr | 21.5 | | nr | nr | nr | nr | 29.7 | nr | nr | | nr | |
| 269 | 19.4 | nr | | nr | | 17.6 | | nr | 18.2 | | 12.1 | nr | nr | nr | nr | 33.1 | nr | | nr | |
| 274 | 21.3 | nr | | nr | | nr | | 20.5 | nr | | 12.4 | 12.4 | 22.4 | nr | nr | nr | nr | | nr | |
| 277 | nr | nr | | 18.6 | | nr | | nr | nr | | nr | 13.6 | 23.5 | 23.1 | nr | nr | nr | | nr | |
| 280 | nr | 20.7 | | 21.2 | | nr | | nr | nr | | 12.7 | nr | 23.5 | 23.1 | nr | nr | nr | | nr | |
| 296 | nr | 22.4 | | nr | | 20.7 | | 21.0 | 20.7 | | nr | 12.8 | 22.8 | nr | nr | nr | nr | | nr | |
| 303 | nr | nr | | nr | | 20.7 | | nr | nr | | nr | nr | nr | nr | nr | nr | nr | | nr | |
| 307 | nr | nr | | nr | | nr | | nr | nr | | nr | nr | nr | nr | nr | 29.4 | nr | | nr | |
| 308 | 19.4 | 19.2 | | 17.4 | | 17.5 | | 19.2 | nr | | nr | 11.8 | 20.0 | 19.8 | nr | 21.9 | nr | | nr | |
| 311 | nr | 21.3 | | 19.4 | | nr | | 19.8 | 20.1 | | 12.9 | 13.5 | nr | 22.6 | 31.8 | 31.3 | nr | | nr | |
| 318 | 21.6 | 22.1 | | nr | | 18.8 | | nr | 19.4 | | nr | nr | nr | 20.0 | 31.3 | 31.5 | nr | | nr | |
| 319 | 20.7 | 19.9 | | nr | | 17.2 | | 19.1 | 19.1 | | 12.7 | 12.8 | nr | 21.1 | 29.0 | nr | nr | | 9.1 | |
| 325 | 19.8 | 18.6 | | nr | | nr | | nr | 25.1 | | nr | Nr | nr | nr | nr | nr | nr | | nr | |
| 330 | 21.4 | 21.2 | | nr | | 18.2 | | 21.3 | 20.3 | | 12.3 | Nr | nr | 22.3 | 27.4 | 26.8 | nr | | nr | |
| 343 | nr | nr | | nr | | nr | | nr | nr | | 13.8 | Nr | nr | nr | 31.3 | nr | nr | | nr | |
| 346 | 21.3 | 20.7 | | 18.4 | | 18.3 | | 19.6 | 19.7 | | 12.8 | 12.5 | nr | 22.1 | nr | 26.0 | nr | | nr | |
| 356 | 19.2 | 18.9 | | 16.8 | | 16.1 | | nr | 18.6 | | 12.5 | 12.6 | 22.2 | 21.9 | nr | 28.3 | nr | | nr | |
| 362 | nr | nr | | nr | | 20.1 | | nr | nr | | nr | 11.6 | nr | nr | 28.0 | nr | nr | | nr | |
| **Mean value** | 20.8 | 20.7 | | 18.6 | | 18.4 | | 20.3 | 20.3 | | 12.9 | 12.7 | 22.8 | 21.9 | 29.2 | 28.1 | nr | | 9.1 | |
| **LATERALITY INDEX** | | | | | | | | | | | | | | | | | | | | |
| **SU** | **LENGTH MEASUREMENTS** | | | | | | | | | | **SECTION MEASUREMENTS** | | | | | | | | | |
|  | **HUMERUS** | | **ULNA** | | **RADIUS** | | **FEMUR** | | | **TIBIA** | **HUMERUS** | | **ULNA** | | **RADIUS** | | **FEMUR** | | **TIBIA** | |
| 110.0 | nr | | nr | | nr | | nr | | | nr | nr | | nr | | 2.2 | | nr | | nr | |
| 147 | nr | | nr | | nr | | nr | | | nr | nr | | nr | | 2.1 | | -1.0 | | nr | |
| 158.0 | -0.3 | | nr | | nr | | nr | | | nr | 3.9 | | nr | | nr | | -2.6 | | nr | |
| 169.0 | nr | | nr | | nr | | 1.7 | | | nr | nr | | nr | | nr | | -2.2 | | nr | |
| 173.0 | 1.4 | | nr | | 3.8 | | -0.7 | | | -9.0 | 1.6 | | nr | | -5.3 | | 6.4 | | -0.2 | |
| 176.0 | nr | | nr | | nr | | nr | | | nr | 0.9 | | nr | | 3.5 | | nr | | 1.5 | |
| 185.0 | nr | | nr | | nr | | nr | | | nr | 8.9 | | 2.0 | | nr | | nr | | nr | |
| 189.0 | nr | | nr | | nr | | nr | | | 0.9 | nr | | 8.3 | | 2.1 | | -2.2 | | 3.9 | |
| 204.0 | 1.1 | | 1.3 | | nr | | -1.5 | | | nr | 4.1 | | 4.4 | | nr | | 0.4 | | -0.1 | |
| 221.0 | 1.8 | | 0.3 | | -0.5 | | nr | | | nr | 0.5 | | -3.3 | | 0.2 | | nr | | nr | |
| 231.0 | nr | | nr | | nr | | nr | | | nr | nr | | nr | | 2.1 | | nr | | nr | |
| 239.0 | 0.6 | | 1.1 | | nr | | -2.5 | | | -0.3 | 7.2 | | 0.0 | | 6.1 | | 3.2 | | 3.3 | |
| 245.0 | nr | | nr | | 0.7 | | nr | | | nr | 3.5 | | nr | | 1.0 | | -1.4 | | nr | |
| 249.0 | nr | | nr | | nr | | nr | | | nr | nr | | nr | | nr | | nr | | -1.2 | |
| 274.0 | nr | | nr | | nr | | -1.3 | | | nr | nr | | nr | | nr | | -1.1 | | 2.7 | |
| 277.0 | nr | | nr | | nr | | nr | | | 0.1 | nr | | nr | | -1.3 | | nr | | 1.1 | |
| 280.0 | nr | | nr | | nr | | 0.2 | | | 0.1 | nr | | nr | | nr | | -0.5 | | 1.1 | |
| 296.0 | nr | | nr | | 2.2 | | nr | | | nr | 0.6 | | -3.3 | | 3.2 | | -2.0 | | -0.5 | |
| 303.0 | nr | | nr | | nr | | nr | | | nr | 0.3 | | -3.3 | | 1.2 | | 0.3 | | nr | |
| 308.0 | 2.3 | | 3.3 | | nr | | nr | | | nr | 2.9 | | 2.4 | | -0.3 | | 5.7 | | 1.8 | |
| 311.0 | nr | | nr | | nr | | nr | | | nr | 3.3 | | nr | | -1.5 | | -3.6 | | 1.9 | |
| 318.0 | nr | | nr | | nr | | nr | | | nr | -1.2 | | nr | | nr | | nr | | -0.3 | |
| 319.0 | 0.7 | | nr | | nr | | nr | | | 2.2 | 4.2 | | nr | | 2.2 | | -0.7 | | 10.7 | |
| 320.0 | nr | | nr | | nr | | nr | | | nr | nr | | nr | | nr | | nr | | nr | |
| 325.0 | nr | | nr | | nr | | nr | | | nr | 8.8 | | 2.5 | | nr | | 0.0 | | 4.5 | |
| 330.0 | 1.5 | | nr | | nr | | nr | | | nr | 4.3 | | nr | | 3.9 | | 0.8 | | nr | |
| 343.0 | nr | | nr | | nr | | nr | | | nr | nr | | nr | | nr | | nr | | 13.5 | |
| 346.0 | -0.2 | | -0.1 | | nr | | -0.5 | | | nr | 2.6 | | 0.3 | | -0.3 | | 2.1 | | nr | |
| 356.0 | 0.2 | | 1.5 | | nr | | 0.6 | | | -2.3 | 3.1 | | 5.6 | | 3.6 | | -0.6 | | -6.4 | |
| **Mean value** | 0.9 | | 1.2 | | 1.6 | | -0.5 | | | -1.2 | 3.3 | | 1.4 | | 1.4 | | 0.1 | | 2.2 | |
