# Supplementary material for: Who were the miners of Allumiere? A multidisciplinary approach to reconstruct the osteobiography of an Italian worker community
Source: PLoS One. 2018 Oct 11;13(10):e0205362. doi: 10.1371/journal.pone.0205362 (PMC6181348; doi:10.1371/journal.pone.0205362)
Supplement: S4 Table — Neoplasia (NP), Infectious diseases (periostitis, PO; osteomyelitis, OM; infections, IN), trauma and stress markers (fractures, FR; grasping GR), degenerative pathologies (degenerative diseases, DG; and axial degereative diseases (Schmörl’s nodes, SN)) and congenital disorders (CO), and inflammation (IF). (DOCX) [file pone.0205362.s004.docx]

**S4 Table. Presence of pathology/stress markers for each individual.**

Neoplasia (NP), Infectious diseases (periostitis, PO; osteomyelitis, OM; infections, IN), degenerative pathologies (degenerative diseases, DG; and axial degenerative diseases -Schmörl’s nodes, SN-), congenital disorders (CO), inflammation (IF), and trauma and stress markers (fractures, FR; grasping GR).

| **SU** | **Sex** | **Age** | **Neoplasia** | **Infectious diseases** | | | **Degerative pathologies** | | **Congenital disorders** | **Inflammation** | **Trauma and stress markers** | |
| --- | --- | --- | --- | --- | --- | --- | --- | --- | --- | --- | --- | --- |
|  |  |  | **NP** | **PO** | **OM** | **IN** | **DG** | **SN** | **CO** | **IF** | **FR** | **GR** |
| **110** | **M** | **41-50** |  |  |  |  | x | x |  |  | x |  |
| **135** | **M** | **31-40** |  | x |  |  | x |  |  |  |  |  |
| **139** | **M** | **41-50** |  |  |  |  | x | x |  |  | x |  |
| **144** | **M** | **31-40** | x |  |  | x | x | x |  |  |  |  |
| **147** | **M** | **19-30** |  |  | x |  | x | x |  |  | x | x |
| **158** | **M** | **31-40** |  | x |  |  | x | x |  |  | x |  |
| **159** | **M** | **31-40** |  |  |  |  | x |  |  |  |  |  |
| **169** | **M** | **19-30** |  |  |  |  | x | x |  |  |  | x |
| **173** | **M** | **19-30** |  |  |  |  | x | x |  |  | x | x |
| **176** | **M** | **19-30** |  |  |  |  | x |  |  |  |  |  |
| **179** | **M** | **31-40** |  |  |  |  |  |  |  |  |  |  |
| **182** | **IND** | **31-40** |  |  |  |  | x | x |  |  |  |  |
| **185** | **M** | **51-60** |  | x |  |  | x | x |  |  |  |  |
| **189** | **M** | **19-30** |  | x |  |  | x | x |  |  | x | x |
| **192** | **M** | **19-30** |  |  |  |  | x | x |  |  |  |  |
| **195** | **M** | **IA** |  |  |  |  | x | x |  |  |  |  |
| **198** | **M** | **31-40** |  |  |  |  | x | x |  |  | x |  |
| **201** | **M** | **31-40** |  | x | x | x | x | x |  | x |  | x |
| **204** | **M** | **31-40** |  |  |  |  | x | x |  |  |  | x |
| **213** | **IND** | **31-40** |  |  |  |  | x | x |  |  |  | x |
| **216** | **F** | **41-50** |  |  |  |  | x | x |  | x | x | x |
| **221** | **M** | **31-40** |  | x | x | x | x | x |  |  | x |  |
| **231** | **M** | **41-50** |  | x |  |  | x | x |  |  | x |  |
| **234** | **F** | **31-40** |  | x | x | x | x | x |  |  |  | x |
| **239** | **M** | **19-30** |  |  |  |  | x | x |  |  | x |  |
| **245** | **M** | **51-60** |  |  |  |  | x | x |  |  | x |  |
| **249** | **M** | **19-30** |  |  |  |  | x |  |  | x |  |  |
| **256** | **M** | **19-30** |  |  |  |  |  | x |  |  |  |  |
| **269** | **M** | **19-30** |  | x | x |  | x | x |  |  |  | x |
| **270** | **F** | **19-30** |  |  |  |  | x | x |  |  |  |  |
| **272** | **M** | **31-40** |  |  |  |  |  |  |  |  |  |  |
| **274** | **M** | **19-30** | x | x |  | x | x | x |  |  |  | x |
| **277** | **M** | **41-50** |  |  |  |  | x | x |  |  | x | x |
| **280** | **M** | **41-50** | x |  |  | x | x |  |  |  |  | x |
| **290** | **M** | **41-50** |  |  |  |  | x | x |  |  | x |  |
| **293** | **F** | **19-30** |  |  |  | x | x | x |  |  |  | x |
| **296** | **M** | **51-60** |  | x |  |  | x | x |  |  |  | x |
| **303** | **M** | **19-30** |  | x |  |  | x | x |  |  |  | x |
| **307** | **M** | **31-40** |  |  |  |  | x | x |  |  |  |  |
| **308** | **M** | **19-30** | x |  |  |  | x | x | x | x | x | x |
| **311** | **M** | **19-30** |  | x |  |  | x | x |  |  |  |  |
| **318** | **M** | **19-30** |  | x | x |  | x | x |  |  |  |  |
| **319** | **M** | **19-30** |  |  | x |  | x | x |  | x |  |  |
| **320** | **M** | **31-40** |  |  |  |  | x |  |  |  |  |  |
| **324** | **NR** | **IA** |  |  |  |  |  |  |  |  |  |  |
| **325** | **M** | **31-40** |  |  |  |  | x | x |  |  |  | x |
| **330** | **M** | **41-50** | x | x |  |  | x | x |  |  |  |  |
| **339** | **F** | **19-30** |  |  |  |  | x |  |  |  |  |  |
| **343** | **M** | **31-40** |  | x |  | x | x | x |  |  |  | x |
| **346** | **M** | **41-50** |  |  |  |  | x | x |  |  |  | x |
| **356** | **M** | **31-40** |  | x |  |  | x | x |  | x |  |  |
| **359** | **M** | **31-40** |  |  |  |  |  |  |  |  |  |  |
| **362** | **M** | **31-40** |  | x |  |  | x | x |  |  |  | x |
| **Total** | | | 5 | 18 | 7 | 8 | 48 | 42 | 1 | 6 | 15 | 21 |
| **(percentage value)** | | | 9% | 32% | 13% | 14% | 86% | 75% | 2% | 11% | 27% | 38% |
