# Supplementary material for: Who were the miners of Allumiere? A multidisciplinary approach to reconstruct the osteobiography of an Italian worker community
Source: PLoS One. 2018 Oct 11;13(10):e0205362. doi: 10.1371/journal.pone.0205362 (PMC6181348; doi:10.1371/journal.pone.0205362)
Supplement: S5 Table — (DOCX) [file pone.0205362.s005.docx]

**Table S5. Results of the analysis on degenerative disease pattern (nr: not recordable).**

| **US** | **Sex** | **Age** | **vertebrae** | **shoulder** | **elbow** | **wrist** | **hand** | **hip** | **knee** | **ankle** | **foot** |
| --- | --- | --- | --- | --- | --- | --- | --- | --- | --- | --- | --- |
| **110** | **M** | **41-50** |  | x | x | x |  |  |  |  |  |
| **135** | **M** | **31-40** | x |  |  | x |  | x |  |  |  |
| **139** | **M** | **41-50** | x | x |  |  |  |  |  | x | x |
| **144** | **M** | **31-40** | x | x |  |  |  |  |  |  |  |
| **147** | **M** | **19-30** | x | x | x |  |  | x |  |  |  |
| **158** | **M** | **31-40** | x | x | x |  |  | x |  |  |  |
| **159** | **M** | **31-40** |  | x |  |  |  |  |  |  |  |
| **169** | **M** | **19-30** |  | x |  | x |  |  |  |  |  |
| **173** | **M** | **19-30** |  |  |  | x |  |  |  |  |  |
| **176** | **M** | **19-30** |  | x | x |  |  | x |  |  |  |
| **179** | **M** | **31-40** | nr | nr | nr | nr | nr | nr | nr | nr | nr |
| **185** | **M** | **51-60** | x | x | x | x |  | x |  |  |  |
| **189** | **M** | **19-30** | x | x |  | x |  | x | x |  |  |
| **192** | **M** | **19-30** |  | x |  |  |  | x |  |  |  |
| **195** | **M** | **IA** | x | x |  |  |  |  |  |  |  |
| **198** | **M** | **31-40** |  |  | x | x |  | x |  | x |  |
| **201** | **M** | **31-40** |  | x |  | x |  | x | x |  |  |
| **204** | **M** | **31-40** | x |  | x | x |  |  |  |  |  |
| **221** | **M** | **31-40** | x | x | x | x |  | x | x |  |  |
| **231** | **M** | **41-50** | x | x | x | x |  | x |  |  |  |
| **239** | **M** | **19-30** |  |  |  |  |  |  |  |  |  |
| **245** | **M** | **51-60** | x | x |  | x | x | x | x | x | x |
| **249** | **M** | **19-30** |  |  |  |  |  |  |  |  |  |
| **256** | **M** | **19-30** | nr | nr | nr | nr | nr | nr | nr | nr | nr |
| **269** | **M** | **19-30** |  |  | x | x |  | x |  |  |  |
| **272** | **M** | **31-40** | nr | nr | nr | nr | nr | nr | nr | nr | nr |
| **274** | **M** | **19-30** |  |  | x | x |  | x |  |  |  |
| **277** | **M** | **41-50** | x | x | x |  |  | x | x |  |  |
| **280** | **M** | **41-50** |  |  | x |  |  | x |  |  |  |
| **290** | **M** | **41-50** | x | x | x |  |  |  |  |  |  |
| **296** | **M** | **51-60** | x | x | x | x |  | x |  |  |  |
| **303** | **M** | **19-30** | x | x | x |  |  | x |  | x |  |
| **307** | **M** | **31-40** |  | x |  |  |  | x |  |  |  |
| **308** | **M** | **19-30** |  | x | x |  |  | x |  |  |  |
| **311** | **M** | **19-30** |  | x |  | x |  | x |  |  |  |
| **318** | **M** | **19-30** |  | x |  |  |  |  |  | x | x |
| **319** | **M** | **19-30** |  | x |  |  |  | x |  |  | x |
| **320** | **M** | **31-40** |  |  |  |  |  | x |  |  |  |
| **325** | **M** | **31-40** |  | x |  |  |  | x |  |  |  |
| **330** | **M** | **41-50** | x | x |  | x |  | x |  |  |  |
| **343** | **M** | **31-40** |  | x |  |  |  | x |  | x |  |
| **346** | **M** | **41-50** |  | x |  |  |  | x |  |  |  |
| **356** | **M** | **31-40** | x |  |  | x |  |  |  |  |  |
| **359** | **M** | **31-40** |  |  |  |  |  |  |  |  |  |
| **362** | **M** | **31-40** |  | x |  |  |  |  |  |  |  |
