# Supplementary material for: Who were the miners of Allumiere? A multidisciplinary approach to reconstruct the osteobiography of an Italian worker community
Source: PLoS One. 2018 Oct 11;13(10):e0205362. doi: 10.1371/journal.pone.0205362 (PMC6181348; doi:10.1371/journal.pone.0205362)
Supplement: S6 Table — (DOCX) [file pone.0205362.s006.docx]

**S6 Table.** The molecules identified in dental calculus by GC-MS analysis were listed and clustered in biochemical classes for each sample

| **SU 110** | | |
| --- | --- | --- |
| Sugars | Milk sugars | alpha.-D-Galactopyranose, 2-(acetylamino)-2-deoxy-  L(-)-Fucose, tetramethyl ether |
| Amino acids |  | D-Asparagine |
| Fatty acids | ω-6 | 9,12-Octadecadienoic acid, methyl ester, (E,E)-  8,11,14-Eicosatrienoic acid, methyl ester, (Z,Z,Z)- |
|  | others | Decanoic acid, methyl ester $$ Capric acid methyl ester  Tetradecanoic acid, 12-methyl-, methyl ester  Cyclopentaneundecanoic acid, methyl ester  Heptacosanoic acid, methyl ester  Hexanoic acid, 3-ethyl-, methyl ester  Hexadecanoic acid, 15-methyl-, methyl ester  Docosanoic acid, methyl ester $$ Behenic acid, methyl ester  Pentadecanoic acid, 14-methyl-, methyl ester  13-Docosenoic acid, methyl ester |
| Alcaloids and derivatives |  | Theophylline, 7-(2-hydroxy-3-(N-(2-hydroxyethyl)-N-methylamino)propyl)-  Securinine  dl-Stachydrine |
| Terpenes and derivatives |  | 1,2-Dihydrolinalool  Isocitronellol |
| Others vegetal and non-vegetal marker |  | Homogentisic acid, 3TBDMS derivative  Betaine $$ .alpha.-Earleine  Isothiocyanatoacetaldehyde dimethyl acetal  2(3H)-Furanone, 5-ethyldihydro- $$ .gamma.-Caprolactone |

| **SU 147** | | |
| --- | --- | --- |
| Amino acids |  | isoasparagina  L-Proline, ethyl ester |
| Fatty acids | ω-6 | 9,12-Octadecadienoic acid, methyl ester, (E,E)- |
|  | others | Decanoic acid, methyl ester  Tridecanoic acid, methyl ester  Methyl valerate $$ Pentanoic acid, methyl ester  Tetradecanoic acid, 12-methyl-, methyl ester  Hexadecanoic acid, methyl ester $$ Palmitic acid, methyl ester  13-Docosenoic acid, methyl ester  Heptacosanoic acid, methyl ester  11-Octadecenoic acid, methyl ester  Docosanoic acid, methyl ester $$ Behenic acid, methyl ester  Heneicosanoic acid, methyl ester |
| Alcohols |  | 2-Nonanol  trans-2-Undecen-1-ol |
| Others vegetal and non-vegetal marker |  | Succinic acid, hept-2-yl 2-ethoxyethyl ester  Desulfoglucobrassicin  Malonic acid ester |
| Combustion marker |  | 1-Naphthalenecarboxaldehyde  Benzene, [(1,2-dimethyl-2-propenyl)oxy]- |
| **SU 169** | | |
| Amino acids |  | DL-Asparagine  Serine |
| Fatty acids | ω-6 | 9,12-Octadecadienoic acid, methyl ester, (E,E)- |
|  | others | Pentanoic acid, 4-methyl-, methyl ester  Heptacosanoic acid, methyl ester  Pentadecanoic acid, 14-methyl-, methyl ester  11-Octadecenoic acid, methyl ester  13-Docosenoic acid, methyl ester  Docosanoic acid, methyl ester $$ Behenic acid, methyl ester  Eicosanoic acid, methyl ester  Hexanoic acid, anhydride |
| Alcohols |  | 3-Nonen-1-ol, (Z)- |
| Alcaloids and derivatives |  | Theophylline, 7-(2-hydroxy-3-(N-(2-hydroxyethyl)-N-methylamino)propyl)- |
| Terpenes and derivatives |  | Limonene |
| Others vegetal and non-vegetal marker |  | Isothiocyanatoacetaldehyde dimethyl acetal  Malonic acid, dihydroxy-, diisobutyl ester  Betaine $$ .alpha.-Earleine |
| **SU 173** | | |
| Sugars |  | cis-Inositol  D-Glucitol, 1,4:3,6-dianhydro- $$ D-Sorbitol, 1,4:3,6-dianhydro |
| Amino acids |  | DL-Asparagine |
| Fatty acids | ω-6 | 9,12-Octadecadienoic acid, methyl ester, (E,E)- |
|  | others | Butanoic acid, 3-methyl-, methyl ester $$ Isovaleric acid, methyl ester  Tetradecanoic acid, 12-methyl-, methyl ester  Cyclopentaneundecanoic acid, methyl ester  Heptacosanoic acid, methyl ester  Hexanoic acid, 3-ethyl-, methyl ester  Pentanoic acid, 4-methyl-, methyl ester $$ Valeric acid, 4-methyl-, methyl ester  13-Docosenoic acid, methyl ester  Decanoic acid, methyl ester $$ Capric acid methyl ester  Docosanoic acid, methyl ester $$ Behenic acid, methyl ester |
| Alcaloids and derivatives |  | Theophylline, 7-(2-hydroxy-3-(N-(2-hydroxyethyl)-N-methylamino)propyl)- |
| Others vegetal and non-vegetal marker |  | Malonic acid, dihydroxy-, diisobutyl ester  Desulfoglucobrassicin  Isothiocyanatoacetaldehyde dimethyl acetal  Betaine $$ .alpha.-Earleine |
| **SU 176** | | |
| Sugars | Milk sugars | D-Galactose, 2,3,4,5-tetra-O-methyl- |
|  | others | Myo-Inositol, 4-C-methyl- |
| Amino acids |  | DL-Asparagine  Serine  dl-Citrulline  Valine  L-Alanine, N-dimethylaminomethylene- |
| Fatty acids | ω-6 | 9,12-Octadecadienoic acid, methyl ester, (E,E)- |
|  | others | Decanoic acid, methyl ester  Octanoic acid, methyl ester  Tetradecanoic acid, 12-methyl-, methyl ester  Pentanoic acid, 4-methyl-, methyl ester  Hexadecanoic acid, methyl ester $$ Palmitic acid, methyl ester  Undecanoic acid, 11-bromo-, methyl ester  Tridecanoic acid, methyl ester  11-Octadecenoic acid, methyl ester  Heptacosanoic acid, methyl ester  Docosanoic acid, methyl ester $$ Behenic acid, methyl ester  Eicosanoic acid, methyl ester |
| Alcohols |  | 1-Heptanol, 2,4-dimethyl-, |
| Alcaloids and derivatives |  | Theophylline, 7-(2-hydroxy-3-(N-(2-hydroxyethyl)-N-methylamino)propyl)-  Conhydrin  Acetyl turicine |
| Terpenes and derivatives |  | Limonene |
| Others vegetal and non-vegetal marker |  | Isothiocyanic acid, propyl ester  cis-.beta.-Methyl-.gamma.-Octalactone  Desulfoglucobrassicin  Betaine $$ .alpha.-Earleine  Pentanal, 2,3-dimethyl- $$ Valeraldehyde, 2,3-dimethyl- |
| **SU 185** | | |
| Sugars | Milk sugars | .alpha.-D-Galactopyranose, 2-(acetylamino)-2-deoxy- |
|  | others | 3,4-Anhydro-d-galactosan  Phenyl-.beta.-D-glucoside $$.beta.-D-Glucopyranoside, phenyl |
| Amino acids |  | L-Alanine, N-dimethylaminomethylene-  Arginine |
| Fatty acids | ω-6 | 9,12-Octadecadienoic acid, methyl ester, (E,E)- |
|  | others | Tetradecanoic acid, 12-methyl-, methyl ester  Stearic acid hydrazide $$ Octadecanoic acid, hydrazide  Heptacosanoic acid, methyl ester  Eicosanoic acid, methyl ester  Cyclopropanepentanoic acid, 2-undecyl-, methyl ester, trans-  Cyclopentaneundecanoic acid, methyl ester  Octanoic acid, methyl ester  Docosanoic acid, methyl ester $$ Behenic acid, methyl ester  Heptacosanoic acid, methyl ester  Decanoic acid, methyl ester  Pentanoic acid, 4-methyl-, methyl ester $$ Valeric acid, 4-methyl-, methyl ester  13-Docosenoic acid, methyl ester  Tridecanoic acid, methyl ester  Triacontanoic acid, methyl ester  Hexadecanoic acid, 15-methyl-, methyl ester  Oleic Acid $$ 9-Octadecenoic acid (Z)- |
| Alcohols |  | (Z)-4-Decen-1-ol  1-Octanol  2-Nonen-1-ol, (E)- |
| Alcaloids and derivatives e derivati |  | Theophylline, 7-(2-hydroxy-3-(N-(2-hydroxyethyl)-N-methylamino)propyl)- |
| Terpenes and derivatives |  | Limonene  1-Octanol, 3,7-dimethyl-, (S)- $$ (S)-Dihydrocitronellol  3-Octanol, 3,7-dimethyl- $$ Linalool tetrahydride |
| Others vegetal and non-vegetal marker |  | Isothiocyanatoacetaldehyde dimethyl acetal  1,5-Heptadien-4-one, 3,3,6-trimethyl- $$ Artemisia ketone  1,5-Heptadien-4-ol, 3,3,6-trimethyl- $$ Artemisia alcol  Isocyanic acid, ethyl ester  Sorbic acid vinyl ester  Malonic acid, dihydroxy-, diisobutyl ester  Octanal, 7-methoxy-3,7-dimethyl- $$ Methoxycitronellal  Pentanal, 2,4-dimethyl- $$ Valeraldehyde, 2,4-dimethyl-  Cholesterol 3-O-[[2-acetoxy]ethyl]- |
| **SU 192** | | |
| Sugars |  | Rhamnose $$ L-Mannose, 6-deoxy- $$ Isodulcitol |
| Amino acids |  | Lysine  Serine  dl-Proline |
| Fatty acids | ω-6 | 9,12-Octadecadienoic acid, methyl ester, (E,E)- |
|  | others | Pentanoic acid, 4-methyl-, methyl ester  Hexanoic acid, anhydride $$ Caproic acid anhydride  Hexadecanoic acid, 15-methyl-, methyl ester  Decanoic acid, methyl ester $$ Capric acid methyl ester  Stearic acid hydrazide $$ Octadecanoic acid, hydrazide  13-Docosenoic acid, methyl ester  Heptacosanoic acid, methyl ester  Dodecanoic acid, 2,3-bis(acetyloxy)propyl ester  Docosanoic acid, methyl ester $$ Behenic acid, methyl ester  Eicosanoic acid, methyl ester $$ Methyl arachisate |
| Alcohols |  | (Z)-4-Decen-1-ol  1-Heptanol, 2,4-dimethyl-  1-Octanol, 2-nitro- |
| Alcaloids and derivatives e derivati |  | Methylephedrine |
| Terpenes and derivatives |  | (-)-Limonene |
| Others vegetal and non-vegetal marker |  | Isothiocyanatoacetaldehyde dimethyl acetal  cis-.beta.-Methyl-.gamma.-Octalactone  2(3H)-Furanone, dihydro-5-methyl- $$ .gamma.-Pentalactone  Betaine $$ .alpha.-Earleine  Pentanal, 3-methyl- $$ Valeraldehyde, 3-methyl-  Octanal, 7-methoxy-3,7-dimethyl- $$ Methoxycitronellal |
| **SU 198** | | |
| Sugars | Milk sugars | .alpha.-D-Galactopyranose, 2-(acetylamino)-2-deoxy-  L-(-)-Fucose, tetrakis(trifluoroacetate), benzyloxime (isomer 1) |
|  | others | 3,4-Anhydro-d-galactosan  D-(-)-Fructose, pentaacetate, benzyloxime (isomer 2)  D-Glucitol, 1,4:3,6-dianhydro- $$ D-Sorbitol, 1,4:3,6-dianhydro |
| Amino acids |  | L-Alanine, N-dimethylaminomethylene-  l-Isoasparagine |
| Fatty acids | ω-6 | 8,11,14-Eicosatrienoic acid, methyl ester, (Z,Z,Z)-  9,12-Octadecadienoic acid, methyl ester, (E,E)- |
|  | others | Decanoic acid, methyl ester  Butanoic acid, 3-methyl-, methyl ester  Pentanoic acid, 4-methyl-, methyl ester  Docosanoic acid, methyl ester $$ Behenic acid, methyl ester  Pentadecanoic acid, 14-methyl-, methyl ester  Triacontanoic acid, methyl ester $$ Methyl melissate  Eicosanoic acid, methyl ester $$ Methyl arachisate  6-Octadecenoic acid, methyl ester, (Z)-  15-Tetracosenoic acid, methyl ester  Octanoic acid, methyl ester  Octadecanoic acid, methyl ester $$ Stearic acid, methyl ester |
| Alcohols |  | Phytol  1-Heptanol, 2-propyl-  1-Octanol, 2,2-dimethyl- |
| Terpenes and derivatives |  | Limonene oxide, trans-  Fenchol, exo-  Borneol, pentamethyldisilanyl ether  Linalool tetrahydride $$ Tetrahydrolinalool  Cumene hydroperoxide, TMS derivative |
| Phenolic compounds and derivatives |  | 1H-Cyclopentano[c]coumarine, 2,3-dihydro-7-benzyloxy-  3,5-di-tert-Butylcatechol |
| Others vegetal and non-vegetal marker |  | Artemisia ketone $$ Isoartemisia ketone  2,6-Lupetidine  Homogentisic acid, 3TBDMS derivative  Betaine $$ .alpha.-Earleine  Octanal, 7-methoxy-3,7-dimethyl- $$ Methoxycitronellal  Cucumber aldehyde  Cholesterol 3-O-[[2-acetoxy]ethyl]-  7-Dehydrocholesterol isocaproate |
| Combustion marker |  | Cyclopenta[a]naphthalene, 5-methyl-1,2,3,9b-tetraaza- |
| **SU 204** | | |
| Sugars | Milk sugars | D-Galactose, 2,3,4,5-tetra-O-methyl- |
|  | others | 3,4-Anhydro-d-galactosan |
| Amino acids |  | DL-Asparagine  Serine |
| Fatty acids | ω-6 | 9,12-Octadecadienoic acid, methyl ester, (E,E)- |
|  | others | 13-Docosenoic acid, methyl ester  Oleic Acid $$ 9-Octadecenoic acid (Z)-  Docosanoic acid, methyl ester $$ Behenic acid, methyl ester  Eicosanoic acid, methyl ester $$ Methyl arachisate  Pentanoic acid, 4-methyl-, methyl ester  Heptacosanoic acid, methyl ester  Stearic acid hydrazide $$ Octadecanoic acid, hydrazide  Tetradecanoic acid, 12-methyl-, methyl ester  Pentadecanoic acid, 14-methyl-, methyl ester  Hexanoic acid, anhydride $$ Caproic acid anhydride |
| Alcaloids and derivatives e derivati |  | Acetyl turicine $$ Acetyl betonicine |
| Terpenes and derivatives |  | Limonene  (+)-Borneol |
| Others vegetal and non-vegetal marker |  | Desulfoglucobrassicin  cis-.beta.-Methyl-.gamma.-Octalactone  Isothiocyanic acid, methyl ester  Betaine $$ .alpha.-Earleine  Octanal, 7-methoxy-3,7-dimethyl- $$ Methoxycitronellal  Pentanal, 2,3-dimethyl- $$ Valeraldehyde, 2,3-dimethyl- |
| **SU 213** | | |
| Sugars | Milk sugars | D-Fucose $$ D-Galactose, 6-deoxy- |
|  |  | beta.-D-Glucopyranoside, methyl 3,6-anhydro- |
| Amino acids |  | Valine |
| Fatty acids | ω-6 | 9,12-Octadecadienoic acid, methyl ester, (E,E)- |
|  | others | Hexanoic acid, anhydride  Stearic acid hydrazide $$ Octadecanoic acid, hydrazide  Pentanoic acid, 4-methyl-, methyl ester  Tetradecanoic acid, 12-methyl-, methyl ester  Pentadecanoic acid, 14-methyl-, methyl ester  Hexanoic acid, 3-ethyl-, methyl ester  11-Octadecenoic acid, methyl ester  Heneicosanoic acid, methyl ester  Docosanoic acid, methyl ester $$ Behenic acid, methyl ester  Eicosanoic acid, methyl ester $$ Methyl arachisate |
| Alcohols |  | 1-Heptanol, 2,4-dimethyl-  1-Octanol, 2,2-dimethyl-  n-Tetracosanol-1 $$ Lignoceric alcohol |
| Alcaloids and derivatives e derivati |  | Methylephedrine |
| Terpenes and derivatives |  | Nerolidol, hexahydro-  Cycloartanol $$ Pollinastanol  Limonene  Linalool |
| Phenolic compounds and derivatives |  | 4,6-di-tert-Butylresorcinol  Pyrogallol, 3TBDMS derivative  3,5-di-tert-Butylcatechol |
| Others vegetal and non-vegetal marker |  | Cholesterol 3-O-[[2-acetoxy]ethyl]- |
| **SU 221** | | |
| Sugars | Milk sugars | d-Galactose oxime  L-(-)-Fucose, tetrakis(trifluoroacetate), benzyloxime (isomer 1)  d-Galactitol, 1-O-octyl- |
| Fatty acids | ω-6 | 9,12-Octadecadienoic acid, methyl ester, (E,E)- |
|  | others | Tetradecanoic acid, 12-methyl-, methyl ester  Stearic acid hydrazide $$ Octadecanoic acid, hydrazide  13-Docosenoic acid, methyl ester  Pentadecanoic acid, 14-methyl-, methyl ester  Heptacosanoic acid, methyl ester  11-Octadecenoic acid, methyl ester  Docosanoic acid, methyl ester $$ Behenic acid, methyl ester |
| Alcohols |  | 1-Heptanol, 2,4-dimethyl-,  1-Octanol, 2,7-dimethyl- |
| Vitamins |  | Vitamin C $$ L-Ascorbic acid $$ Antiscorbutic vitamin |
| Terpenes and derivatives |  | Limonene |
| Others vegetal and non-vegetal marker |  | Isothiocyanic acid, methyl ester  Betaine $$ .alpha.-Earleine  Octanal, 7-methoxy-3,7-dimethyl- $$ Methoxycitronellal |
| **SU 245** | | |
| Sugars |  | d-Mannose $$ Mannose, d- $$ Carubinose  3,4-Anhydro-d-galactosan |
| Amino acids |  | DL-Asparagine  L-Arginine, methyl ester  L-Alanine, N-dimethylaminomethylene-  L-Proline, ethyl ester |
| Fatty acids | ω-6 | 9,12-Octadecadienoic acid, methyl ester, (E,E)- |
|  | others | Butanoic acid, 3-methyl-, 3-methylbutyl ester  Pentanoic acid, 2-methyl- $$ Valeric acid, 2-methyl-  Heptacosanoic acid, methyl ester  13-Docosenoic acid, methyl ester  Cyclopropanepentanoic acid, 2-undecyl-, methyl ester, trans-  Eicosanoic acid, methyl ester  Tetradecanoic acid, 12-methyl-, methyl ester  Cyclopentaneundecanoic acid, methyl ester  Oleic Acid $$ 9-Octadecenoic acid (Z)-  Docosanoic acid, methyl ester $$ Behenic acid, methyl ester |
| Alcaloids and derivatives |  | Theophylline, 7-(2-hydroxy-3-(N-(2-hydroxyethyl)-N-methylamino)propyl)-  Norpseudoephedrine |
| Terpenes and derivatives |  | 1,3,7-Octatriene $$ .alpha.-Ocimene  3-Octanol, 3,7-dimethyl- $$ Linalool tetrahydride |
| Phenolic compounds and derivatives |  | 4,6-di-tert-Butylresorcinol |
| Others vegetal and non-vegetal marker |  | Isothiocyanatoacetaldehyde dimethyl acetal  2,6-Lupetidine $$ Lupetidin  1,5-Heptadien-4-one, 3,3,6-trimethyl- $$ Artemisia ketone  Cholesterol 3-O-[[2-acetoxy]ethyl]-  Octanal, 7-methoxy-3,7-dimethyl- $$ Methoxycitronellal  Pentanal, 2,3-dimethyl- $$ Valeraldehyde, 2,3-dimethyl-  Betaine $$ .alpha.-Earleine |
| **SU 290** | | |
| Fatty acids | ω-6 | 8,11,14-Eicosatrienoic acid, methyl ester, (Z,Z,Z)- |
|  | others | Heptacosanoic acid, methyl ester  Pentadecanoic acid, 14-methyl-, methyl ester  Oleic Acid $$ 9-Octadecenoic acid (Z)-  13-Docosenoic acid, methyl ester |
| Alcohols |  | 1-Hepten-4-ol  dl-Menthol $$ Peppermint camphor |
| Others vegetal and non-vegetal marker |  | 2,6-Lupetidine  2(3H)-Furanone, 5-butyldihydro- $$ .gamma.-Octalactone  Artemisia ketone $$ Isoartemisia ketone  Homogentisic acid, 3TBDMS derivative  1,5-Pentanediamine $$ Animal coniine $$ Cadaverine |
| **SU 303** | | |
| Fatty acids | ω-6 | 9,12-Octadecadienoic acid, methyl ester, (E,E)- |
|  | others | Pentanoic acid, methyl ester $$ Valeric acid, methyl ester  Tetradecanoic acid, 12-methyl-, methyl ester  Heptacosanoic acid, methyl ester  Hexanoic acid, 3-ethyl-, methyl ester  Pentadecanoic acid, 14-methyl-, methyl ester  13-Docosenoic acid, methyl ester  Docosanoic acid, methyl ester $$ Behenic acid, methyl ester  Eicosanoic acid, methyl ester $$ Methyl arachisate |
| Terpenes and derivatives |  | Limonene |
| Others vegetal and non-vegetal marker |  | Betaine $$ .alpha.-Earleine  Octanal, 7-methoxy-3,7-dimethyl- $$ Methoxycitronellal  Cholesterol 3-O-[[2-acetoxy]ethyl]- |
| **SU 304** | | |
| Amino acids |  | Lysine  Serine |
| Fatty acids | others | Butanoic acid, 3-methyl-, methyl ester $$ Isovaleric acid, methyl ester  Heptacosanoic acid, methyl ester  Pentadecanoic acid, 14-methyl-, methyl ester  13-Docosenoic acid, methyl ester  Oleic Acid $$ 9-Octadecenoic acid (Z)-  Docosanoic acid, methyl ester $$ Behenic acid, methyl ester  Pentanoic acid, methyl ester $$ Valeric acid, methyl ester |
| Alcohols |  | 1-Hepten-4-ol |
| Terpenes and derivatives |  | Limonene |
| Others vegetal and non-vegetal marker |  | Isothiocyanatoacetaldehyde dimethyl acetal  Isocyanic acid, ethyl ester  Betaine $$ .alpha.-Earleine  p-Mentha-1(7),2-dien-8-ol |
| **SU 307** | | |
| Fatty acids | ω-6 | 9,12-Octadecadienoic acid, methyl ester, (E,E)- |
|  | others | Pentanoic acid, 4-methyl-, methyl ester  Heptacosanoic acid, methyl ester  Hexanoic acid, 3-ethyl-, methyl ester  Triacontanoic acid, methyl ester $$ Methyl melissate  Docosanoic acid, methyl ester $$ Behenic acid, methyl ester  Hexanoic acid, 3-ethyl-, methyl ester |
| Alcohols |  | 1-Heptanol, 2,4-dimethyl-, |
| Alcaloids and derivatives |  | Theophylline, 7-(2-hydroxy-3-(N-(2-hydroxyethyl)-N-methylamino)propyl)- |
| Others vegetal and non-vegetal marker |  | Desulfoglucobrassicin  Artemisia ketone  Methane, isothiocyanato- $$ Isothiocyanic acid, methyl ester  Octanal, 7-methoxy-3,7-dimethyl- $$ Methoxycitronellal |
| **SU 308** | | |
| Sugars | Milk sugars | .alpha.-D-Galactopyranose, 2-(acetylamino)-2-deoxy- |
|  | others | Allo-Inositol $$ Inositol, allo- |
| Amino acids |  | Lysine |
| Fatty acids | ω-6 | 9,12-Octadecadienoic acid, methyl ester, (E,E)- |
|  | others | Cyclopentaneundecanoic acid, methyl ester  Pentadecanoic acid, 14-methyl-, methyl ester  13-Docosenoic acid, methyl ester  Docosanoic acid, methyl ester $$ Behenic acid, methyl ester  Heptacosanoic acid, methyl ester |
| Alcohols |  | 1-Octanol, 2,7-dimethyl- |
| Others vegetal and non-vegetal marker |  | Betaine $$ .alpha.-Earleine  Octanal, 7-methoxy-3,7-dimethyl- $$ Methoxycitronellal  Allyl Isothiocyanate $$ Isothiocyanic acid, allyl ester |
| Combustion marker |  | Cyclopenta[a]naphthalene, 5-methyl-1,2,3,9b-tetraaza- |
| **SU 318** | | |
| Sugars | Milk sugars | Lactose, .beta.- $$ .beta.-Lactose  2,3-Anhydro-d-galactosan |
|  | others | .beta.-D-Glucopyranoside, methyl 3,6-anhydro- |
| Amino acids |  | Alanine |
| Fatty acids | ω-6 | 9,12-Octadecadienoic acid, methyl ester, (E,E)- |
|  | others | Oleic Acid $$ 9-Octadecenoic acid (Z)-  Docosanoic acid, methyl ester $$ Behenic acid, methyl ester  Hexanoic acid, 3-ethyl-, methyl ester  Heptacosanoic acid, methyl ester  13-Docosenoic acid, methyl ester  Pentanoic acid, methyl ester $$ Valeric acid, methyl ester |
| Alcohols |  | 1-Octanol, 2,7-dimethyl-  (Z)-4-Decen-1-ol |
| Terpenes and derivatives |  | Limonene |
| Phenolic compounds and derivatives |  | Pyrogallol, 3TBDMS derivative |
| Others vegetal and non-vegetal marker |  | Isocyanic acid, isopropyl ester  Malic acid, 3TBDMS derivative  Betaine $$ .alpha.-Earleine  Pentanal, 2,4-dimethyl- $$ Valeraldehyde, 2,4-dimethyl-  Octanal, 7-methoxy-3,7-dimethyl- $$ Methoxycitronellal |
| **SU 319** | | |
| Sugars | Milk sugars | .alpha.-D-Galactopyranose, 2-(acetylamino)-2-deoxy-  Lactose, .beta.- $$ .beta.-Lactose |
| Amino acids |  | Arginine  L-Asparagine  Threonine |
| Fatty acids | ω-6 | 11,14-Eicosadienoic acid, methyl ester  8,11,14-Eicosatrienoic acid, methyl ester, (Z,Z,Z)-  9,12-Octadecadienoic acid, methyl ester, (E,E)- |
|  | others | 15-Tetracosenoic acid, methyl ester  Cyclopentaneundecanoic acid, methyl ester  13-Docosenoic acid, methyl ester  Docosanoic acid, methyl ester $$ Behenic acid, methyl ester  Stearic acid hydrazide $$ Octadecanoic acid, hydrazide  Pentanoic acid, 4-methyl-, methyl ester  Eicosanoic acid, methyl ester  Hexanoic acid, 3-ethyl-, methyl ester  9-Octadecenoic acid (Z)-, hexyl ester  Heptacosanoic acid, methyl ester  Butanoic acid, 4-(dimethylamino)-3-hydroxy-  Tetradecanoic acid, 12-methyl-, methyl ester  Triacontanoic acid, methyl ester $$ Methyl melissate  Pentadecanoic acid, 14-methyl-, methyl ester |
| Alcohols |  | 1-Heptanol, 2-propyl-  1-Octanol, 2-butyl- |
| Phenolic compounds and derivatives |  | Pyrocatechol, 3,5-di-tert-butyl- |
| Others vegetal and non-vegetal marker |  | Artemisia ketone $$ Isoartemisia ketone  cis-.beta.-Methyl-.gamma.-Octalactone  Methane, isothiocyanato- $$ Isothiocyanic acid, methyl ester  Octanal, 7-methoxy-3,7-dimethyl- $$ Methoxycitronellal |
| **SU 330** | | |
| Sugars | Milk sugars | 2-Deoxy-D-galactose |
|  | others | 3,4-Anhydro-d-galactosan  1,2:5,6-Dianhydrogalactitol |
| Amino acids |  | Alanine |
| Fatty acids | ω-6 | 8,11,14-Eicosatrienoic acid, methyl ester, (Z,Z,Z)- |
|  | others | Hexanoic acid, 3-ethyl-, methyl ester  Heptacosanoic acid, methyl ester  Eicosanoic acid, methyl ester $$ Methyl arachisate  Pentanoic acid, 4-methyl-, methyl ester  Tetradecanoic acid, 12-methyl-, methyl ester  n-Hexadecanoic acid $$ Palmitic acid  Stearic acid hydrazide $$ Octadecanoic acid, hydrazide  Triacontanoic acid, methyl ester $$ Methyl melissate  Docosanoic acid, methyl ester $$ Behenic acid, methyl ester |
| Alcaloids and derivatives |  | (-)-Norephedrine |
| Terpenes and derivatives |  | Linalool  Myrcenol  Pinane $$ Dihydropinene  Citronellol |
| **SU 343** | | |
| Sugars | Milk sugars | .alpha.-D-Galactopyranose, 2-(acetylamino)-2-deoxy- |
|  | others | Allo-Inositol |
| Amino acids |  | DL-Asparagine  Serine  Lysine  L-Alanine, N-formyl- |
| Fatty acids | ω-6 | 9,12-Octadecadienoic acid, methyl ester, (E,E)- |
|  | others | Heptacosanoic acid, methyl ester  Eicosanoic acid, methyl ester $$ Methyl arachisate  13-Docosenoic acid, methyl ester  Docosanoic acid, methyl ester $$ Behenic acid, methyl ester  Butanoic acid, 2,3-dimethyl-, methyl ester  Pentanoic acid, 2-methylbutyl ester |
| Others vegetal and non-vegetal marker |  | Acetovanillone, TBDMS derivative  2,6-Lupetidine  Betaine $$ .alpha.-Earleine  1,4-Butanediamine $$ Putrescine |
| **SU 346** | | |
| Sugars |  | 2,3-Anhydro-d-galactosan |
| Amino acids |  | Lysine  Serine |
| Fatty acids | ω-6 | 9,12-Octadecadienoic acid, methyl ester, (E,E)- |
|  | others | Tetradecanoic acid, 12-methyl-, methyl ester  13-Docosenoic acid, methyl ester  Hexanoic acid, 3-ethyl-, methyl ester  Cyclopropanepentanoic acid, 2-undecyl-, methyl ester, trans-  Pentanoic acid, 4-methyl-, methyl ester  Heptacosanoic acid, methyl ester |
| Alcohols |  | 2-Butanol, 3-methyl- |
| Alcaloids and derivatives |  | dl-Stachydrine |
| Terpenes and derivatives |  | cis-Verbenol  (-)-Limonene  3-Octanol, 3,7-dimethyl- $$ Linalool tetrahydride |
| Others vegetal and non-vegetal marker |  | Betaine $$ .alpha.-Earleine  Desulfoglucobrassicin  Artemisia ketone |
| Combustion marker |  | Naphthalene, 1,2,3,4-tetrahydro-1,4-dimethyl- |
| **SU 356** | | |
| Sugars |  | 3,4-Anhydro-d-galactosan  D-Fructose, 3-O-methyl- |
| Amino acids |  | Lysine  L-Alanine, N-formyl- |
| Fatty acids | ω-6 | 9,12-Octadecadienoic acid, methyl ester, (E,E)- |
|  | others | Pentadecanoic acid, 2,6,10,14-tetrame  Stearic acid hydrazide $$ Octadecanoic acid, hydrazide  Decanoic acid, methyl ester $$ Capric acid methyl ester  Cyclopentaneundecanoic acid, methyl ester  Hexanoic acid, 2-propenyl ester  13-Docosenoic acid, methyl ester  Oleic Acid $$ 9-Octadecenoic acid (Z)-  Tetradecanoic acid, 12-methyl-, methyl ester  Hexadecanoic acid, 15-methyl-, methyl ester  Docosanoic acid, methyl ester $$ Behenic acid, methyl ester  Heptacosanoic acid, methyl ester |
| Alcohols |  | 1-Heptanol, 2,4-dimethyl-,  Piperonyl alcohol, TBDMS derivative |
| Alcaloids and derivatives |  | Xanthine, 1,3-dipropyl-8-[4-[.beta.-[(benzyloxycarbonylamino)acetylamino] |
| Terpenes and derivatives |  | 1-Octanol, 3,7-dimethyl- $$ Citronellol, dihydro-  3-Octanol, 3,7-dimethyl- $$ Linalool tetrahydride |
| Others vegetal and non-vegetal marker |  | Pentanal, 2-methyl- $$ Valeraldehyde, 2-methyl-  Octanal, 7-methoxy-3,7-dimethyl- $$ Methoxycitronellal  Betaine $$ .alpha.-Earleine  Malonic acid, dihydroxy-, diisobutyl ester  Homogentisic acid, 3TBDMS derivative |
| Combustion marker |  | Naphthalene, 1,2,3,4-tetrahydro-1,4-dimethyl- |
